# HER2-enriched subtype and novel molecular subgroups drive aromatase inhibitor resistance and an increased risk of relapse in early ER+/HER2+ breast cancer

**DOI:** 10.1016/j.ebiom.2022.104205

**Published:** 2022-08-16

**Authors:** Milana A. Bergamino, Elena López-Knowles, Gabriele Morani, Holly Tovey, Lucy Kilburn, Eugene F. Schuster, Anastasia Alataki, Margaret Hills, Hui Xiao, Chris Holcombe, Anthony Skene, John F. Robertson, Ian E. Smith, Judith M. Bliss, Mitch Dowsett, Maggie C.U. Cheang, POETIC investigators, Abigail Evans, Adrian Ball, Akhil Johri, Ali Nejim, Alison Jones, Allan Corder, Amanda Thorne, Ambika Anand, Amitabha Chakrabarti, Anne Robinson, Anthony Skene, Anupam Modi, Ashraf Patel, Ashutosh Kothari, Brendan McFall, Caroline Mortimer, Caroline Lee, Charlie Chan, Charlotte Abson, Christopher Holcombe, Christopher Hinton, Ciaran Hollywood, Claire Murphy, Clare Crowley, Claudia Harding-Mackean, Clive Griffith, Conrad Lewanski, Daniel Rea, David Hwang, Derek Crawford, Dinesh Thekkinkattil, Douglas Ferguson, Douglas Adamson, Duncan Wheatley, Duraisamy Ravichandran, Ed Babu, Elaine Hyett, Fawzia Ashkanani, Fiona Hoar, Frances Kenny, Gary Dyke, Geoffrey Sparrow, Gilbert, Giles Cunnick, Hafiz Algurafi, Helen Sweetland, Highes-Davies Prof, Hisham Hamed, Ian Smith, Ian Laidlaw, Ilyas Khattak, Jacqueline Newby, Jacqueline Rees-Lee, Jalal Kokan, Jane Barrett, Jay Dolatrai Naik, Jayant Vaidya, Jennifer Forrest, Jitendra Parmar, Jocelyn Adams, John Fox, Jonathan Roberts, Jonathan Dawson, Julie Doughty, Jull Donnelly, Kathleen Dunn, Kian Chin, Kieran Horgan, Kislaya Thakur, Ludger Barthelmes, Lynda Wyld, Madhumita Bhattacharyya, Maher Hadaki, Makam Kishore, Marcus Ornstein, Maria Bramley, Maria Bews-Hair, Marina Parton, Mark Sibbering, Mark Kissin, Mark Churn, Martin Hogg, Mary Quigley, Matthew Hatton, Matthew Winter, Matthew Adelekan, Michael Shere, Michael Carr, Michael Williams, Mohammed Absar, Muhammad Sharif, Muireann Kelleher, Nawaz Walji, Nicholas Williams, Nicholas Gallegos, Nigel Bundred, Olivia Hatcher, Perric Crellin, Peter Crane, Peter Donnelly, Peter Kneeshaw, Philip Walker, Prakash Sinha, Pudhupalayam Bhaskar, Racheal Soulsby, Radha Todd, Raghavan Vidya, Rakesh Mehra, Ramachandran Prasad, Ramsay Cutress, Ravi Sharma, Rebecca Roylance, Rebecca Goranova, Reem Ramzi Salman, Riccardo Bonom, Richard Johnson, Richard Sutton, Rick Linforth, Rob Coleman, Robert Grieve, Robert Leonard, Robert Reichert, Robert Kennedy, Roshan Agarwal, Rozenn Allerton, Russell Burcombe, Ruth Davis, Sankaran Narayanan, Sankaran Chandrasekharan, Sarah Vesty, Seema Seetharam, Serena Ledwidge, Shabana Iqbal, Shamaela Wahee, Shobha Silva, Simon Pain, Simon Holt, Simon Thomson, Simon Smith, Simon Ellenbogen, Simon Holt, Siobhan Laws, Stephen Chan, Stephen Johnston, Steve Holt, Steven Thrush, Stuart McIntosh, Sumohan Chatterjee, Susan Cleator, Tamoor Usman, Tayo Johnson, Tibor Kovacs, Tracey Irvine, Urmila Barthkur, Vanessa Pope, Victoria Alexandra Brown, Vummiti Muralikrishna, Walid Samra, William Maxwell, Zoe Winters

**Affiliations:** a Clinical Trials and Statistics Unit (ICR-CTSU)- Division of Clinical Studies, The Institute of Cancer Research, London, UK; b Royal Marsden Hospital, London, UK; c The Breast Cancer Now Toby Robins Research Centre, The Institute of Cancer Research, London, UK; d Liverpool University Hospitals Foundation Trust, Liverpool, UK; e University Hospitals Dorset NHS-FT, UK; f Faculty of Medicine & Health Sciences, Queen's Medical Centre, Nottingham, UK

**Keywords:** Breast cancer, HER2+, Aromatase inhibitors, HER2-Enriched subtype

## Abstract

**Background:**

Oestrogen receptor positive/ human epidermal growth factor receptor positive (ER+/HER2+) breast cancers (BCs) are less responsive to endocrine therapy than ER+/HER2- tumours. Mechanisms underpinning the differential behaviour of ER+HER2+ tumours are poorly characterised. Our aim was to identify biomarkers of response to 2 weeks’ presurgical AI treatment in ER+/HER2+ BCs.

**Methods:**

All available ER+/HER2+ BC baseline tumours (*n*=342) in the POETIC trial were gene expression profiled using BC360™ (NanoString) covering intrinsic subtypes and 46 key biological signatures. Early response to AI was assessed by changes in Ki67 expression and residual Ki67 at 2 weeks (Ki67_2wk_). Time-To-Recurrence (TTR) was estimated using Kaplan-Meier methods and Cox models adjusted for standard clinicopathological variables. New molecular subgroups (MS) were identified using consensus clustering.

**Findings:**

HER2-enriched (HER2-E) subtype BCs (44.7% of the total) showed poorer Ki67 response and higher Ki67_2wk_ (*p*<0.0001) than non-HER2-E BCs. High expression of *ERBB2* expression, homologous recombination deficiency (HRD) and *TP53* mutational score were associated with poor response and immune-related signatures with High Ki67_2wk_. Five new MS that were associated with differential response to AI were identified. HER2-E had significantly poorer TTR compared to Luminal BCs (HR 2.55, 95% CI 1.14–5.69; *p*=0.0222). The new MS were independent predictors of TTR, adding significant value beyond intrinsic subtypes.

**Interpretation:**

Our results show HER2-E as a standardised biomarker associated with poor response to AI and worse outcome in ER+/HER2+. HRD, *TP53* mutational score and immune-tumour tolerance are predictive biomarkers for poor response to AI. Lastly, novel MS identify additional non-HER2-E tumours not responding to AI with an increased risk of relapse.

**Funding:**

Cancer Research UK (CRUK/07/015).

**Research in context**

**Evidence before this study**

AI treatment is the standard of care and most effective therapy for post-menopausal women with early oestrogen receptor positive (ER+) breast cancer (BC). ER+ BC tumours that also over-express HER2 are very heterogenous with several treatment options but variable responses to the different available drugs. Prior studies have shown that ER+/HER2+ BC show limited antiproliferative response to endocrine therapy and thus, are at a higher risk of recurrence. This may be PgR dependent, as many of those tumours do express low PgR levels. In addition, they have lower response rates to anti-HER2 targeted therapy compared to ER-/HER2+ tumours. Most studies investigating mechanisms of resistance to endocrine therapy have been performed in ER+/HER2- disease and are not well understood in HER2+ BC. Although endocrine-related gene expression has been previously associated with good response to aromatase inhibitors and high levels of *ERBB2* with poor response, there is an overall lack of optimal biomarkers to pair with the optimal treatment for each patient within ER+/HER2+ BC. As such, identifying robust molecular features and defining novel subgroups based on tumour biology is essential to identify the most adequate treatment strategies for this particular BC subgroup.

**Added value of this study**

This study establishes HER2-enriched intrinsic subtype as one of the main components driving poor response to AI and higher risk of relapse in ER+/HER2+ BC. Our results indicate the importance of molecular subtyping of BC beyond the standard HR and HER2 assays. Beyond the intrinsic subtypes, *ERBB2*, DNA damage repair signaling, *p53* mutant surrogate signature and immune-tumour tolerance related signatures are also associated with resistance to treatment.

We also identified five new single gene based molecular subgroups that can distinguish HER2-E and Luminal tumours responding or not to AI treatment and at a higher risk of relapse. Molecular subgroups characterised with high expression of immune related features drive an intrinsic lower risk of relapse despite predicting poor response to AI, while higher levels of *ERBB2* and extracellular matrix related genes lead to worse outcome.

**Implications of all the available evidence**

Firstly, the worse response to treatment and poorer outcome of HER2-enriched BC tumours highlights the potential need of treatment intensification for this intrinsic subtype with additional anti-HER2 targeted therapy, with the limitation, but also “real world” treatment limitation, that not all the patients in the study received the current standard anti-HER2 therapy. The higher sensitivity to aromatase inhibitors and good prognosis associated with luminal tumours, in particular with Luminal A, provides a rational for de-escalation, which has been previously suggested for ER+HER2+ unselected population.

Secondly, the new molecular subgroups show that immune-related features provide ER+/HER2+ BC tumours with an intrinsic good prognosis despite their association with early poor response to AI treatment and might also deserve a de-escalating approach. Furthermore, the assessment of the novel molecular subgroups might be crucial for the identification of some HER2-E BCs patients at a lower risk of relapse and additional non-HER2-E BCs patients with an increased risk of relapse. The combined investigation of the intrinsic subtypes and these new molecular subgroups might be key for the selection of candidate patients for escalated and de-escalated approaches in the future.

Alt-text: Unlabelled box

## Introduction

Human epidermal growth factor receptor 2 positive (HER2+) breast cancer (BC) has been associated with an aggressive phenotype and poor patient outcome.^1^ However, the introduction of HER2-targeted therapies dramatically changed the prognosis of these patients and the natural history of the disease.^2^^,^^3^ Despite the improvement, long-term follow-up data indicate that approximately 15–23% of patients in early stage, still develop recurrent disease.^4^

Fifteen percent of all BC overexpresses *HER2* and approximately 50% of these are also classified as hormone receptor positive (HR+), which confers substantial differences in biology and clinical outcome from HR+/HER2- disease. HR+/HER2+ BCs are molecularly heterogeneous and around 30% of them are HER2-Enriched (HER2-E). This subtype is characterised by a high *HER2/EGFR* pathway activation, increased proliferation and an immune-activated stroma with elevated tumour infiltrating lymphocytes. It has a lower expression of luminal-related genes, than the Luminal A and B subtypes, potentially benefiting greatly from anti-HER2 therapies but poorly from endocrine therapy (ET).^5^, ^6^, ^7^

Resistance to endocrine therapies has been mainly studied in HR+/HER2- BC and includes down-regulation of oestrogen receptor (ER) expression, altered expression of ER co-regulators, presence of ER mutations, ligand-independent activation of ER and co-activators by growth factor receptor kinases.^8^^,^^9^ However, those mechanisms might differ between HER2+ and HER2- tumours, in part due to the differential distribution of intrinsic subtypes within each BC subgroup. HER2-targeted therapies might be felt to negate the importance of development of resistance to AI but while anti-HER2 therapy is generally given for no more than 1 year to primary BC patients, ET is given for at least 5 years. Thus, any residual HER2+ disease after the end of the HER2-targeted therapy remains at risk of an incomplete endocrine response.

The PeriOperative Endocrine-Therapy for Individualised Care (POETIC) trial^10^ is the framework used to study endocrine resistance mechanisms in a large set of ER+/HER2+ BC patients. In the context of POETIC, we hypothesised that resistance mechanisms to ET are driven by baseline genomic features. Gene expression profiles at baseline were assessed and the key genomic characteristics were tested for association with response to AI, as measured by residual levels and changes of Ki67 after two weeks of treatment, and with clinical outcome. We sought to develop predictive signatures of AI response and address the clinical challenge of identifying patients who are likely to benefit from each of the specific therapies.

## Methods

### Patients and samples

All available ER+/HER2+ BC tumours from the POETIC trial in which patients were assigned to 2 weeks of peri-surgical AI or no AI (control) were included in this study.^10^ A consort diagram of the study is shown in Supplementary Figure S1. Ki67 staining of 2-week samples from the control group was restricted to a randomly selected subset due to the minimal expected change on Ki67 from baseline to surgery.^11^ In summary, of 470 ER+/HER2+ patients included in POETIC, we obtained successful results for 342 patients.

### RNA extraction

RNA was extracted from three adjacent macro-dissected 10µm formalin-fixed paraffin-embedded (FFPE) sections from the baseline block of the patients included in the study. The ROCHE High Pure miRNA isolation kit (Roche, Basel, Switzerland) was used following SOP M027 from The Cancer Genome Atlas (TCGA) Program developed by the Biospecimen Core Resource (BCR) at Nationwide Children's Hospital in Columbus, Ohio. Quantification was done using high sensitivity RNA Qubit assays (Thermo Fisher Scientific, Carlsbad, CA).

### Gene expression profiling

Gene expression of 758 genes was assessed using the NanoString nCounter Platform (Nanostring Technologies, Seattle, WA) Breast Cancer 360™ codeset (BC360) covering intrinsic subtypes and 46 key biological signatures (Supplementary Table S1). 150ng of RNA was run and processed on a NanoString nCounter™ FLEX Analysis System according to manufacturer's instructions. NanoString raw data was normalised by NanoString according to the BC360 pipeline using 18 house-keeping genes.

### Immunohistochemistry

ER status was measured locally and was centrally reviewed by immunohistochemistry (IHC). HER2 status was measured locally using IHC and/or fluorescence *in situ* hybridisation (FISH). Ki67 proliferation rate was obtained by IHC in FFPE tissues using the MIB-1 antibody (M7240, DAKO UK, RRID:AB_2631211).^10^^,^^12^ Ki67 has been validated in our laboratory previously and we are part of the International Ki67 in Breast Cancer Working Group.

### Outcomes

The primary endpoints of this study were based on Ki67 as a measure of tumour's resistance to AI. Two Ki67 endpoints were used: 1) Ki67 change was calculated as the difference between Ki67 expression at surgery and baseline (relative change) and was categorised into Ki67 response categories defined as percentage-changes from baseline to surgery: poor response (PR) (reduction <50%), intermediate response (IR) (50–75%) and good response (GR) (>75%). This reflects the antiproliferative response to AI treatment which relates to the treatment benefit. 2) Residual Ki67 at 2-week timepoint (Ki67_2wk_) High (≥ 10%) and Low (<10%) which correlated to the residual risk after AI treatment. The secondary endpoint was time to recurrence (TTR) (local and metastatic recurrence) to evaluate the prognostic significance of the molecular characteristics analysed.

### Statistical analysis

Statistical analysis was performed using the R software (version 3.6.3). P values were considered significant if lower than 0·05. Wilcoxon tests were applied in unpaired comparisons and Kruskall-Wallis tests in all multiple comparisons in both treated and control tumours. Spearman Rank correlation was used to explore the correlation between genes or signatures. Logistic and ordinal regression models were performed to identify signatures significantly associated with 2-week Ki67 and Ki67 response categories respectively. Multiple testing correction was undertaken by the Benjamini & Hochberg (FDR) method.^13^ Significance analysis of microarrays (SAM analysis) was performed in multiclass setting to compare the Ki67 response categories and in the unpaired Two Class to compare extreme response classes (GR and PR) and Ki67_2wk_ High versus Low*.*^14^ Hierarchical clustering of the gene expression profiles that were identified by SAM analysis was also performed.^15^

Consensus clustering was used to identify new molecular subgroups and their association with Ki67 response categories and outcome was tested.^16^ Controls and treated patients were included to obtain the subgroups, but only the treated patients with Ki67 available, were used to assess their predictive value for AI resistance.

TTR was measured as time from randomisation to local, regional, or distant tumour recurrence or death from breast cancer without previous notification of relapse. Second primary cancers and intercurrent deaths were censored. TTR was estimated using Kaplan-Meier methods and Cox models. Multivariable Cox-regression models were adjusted for standard post-surgery clinicopathological variables: grade, tumour size, nodal status and age. We included age as the main driver/surrogate for the adjuvant treatment choice as most patients ≥70 years old did not receive chemotherapy or trastuzumab (67·2%, 82/122) compared to patients <70 years (14%, 31/221). The independent prognostic value of those gene-expression based variables with differential survival in the univariate analysis were assessed. Both controls and treated patients were included in the survival analysis. The assumptions evaluated were: 1. that the dependent variable was ordered; 2. that one or more of the independent variables were continuous, categorical or ordinal; 3. that no multi-collinearity i.e. independent variables were independent from each other and the proportional odds. And 4. that the tests were done for the proportional odds assumptions for the ordinal logistic regression analysis for single gene and signature with the Ki67 Change categories GR/IR/PR. All the assumptions were met without any assumptions’ violations. Patients with missing data were excluded. Only one patient had a missing variable (which constituted only 0·29% of the entire cohort).

### Ethics

The POETIC trial was approved by the London–South East Research Ethics Committee (reference 08/H1102/37) and adopted by the Declaration of Helsinki. Patients provided written informed consent to molecular analysis of their samples for research purposes.

### Role of funding source

Funders did not have any role in study design, data collection, data analysis, interpretation or writing of report.

## Results

### Patient clinicopathological characteristics

In this study, 342 ER+/HER2+ patients with baseline gene expression were included: 237 AI-treated and 105 untreated controls (Supplementary Figure S1). The demographics were well balanced between both groups (Supplementary Table S2). In summary, 93·3% of the tumours were ductal, 48·2% were grade 2 and 38·3% grade 3. At surgery 54·7% had a tumour diameter between 2 and 5 cm and 47·4% had positive nodal status. 60·5 percent of patients received adjuvant chemotherapy and trastuzumab and 98·2% of patients were treated with adjuvant ET.

### PAM50 subtypes and Ki67 endpoints

We evaluated whether intrinsic subtypes could predict response to ET. In the entire subset of patients, 44·7% of tumours classified as HER2-E, 36·3% Luminal B, 17·2% Luminal A and 1·8% Basal-Like. In addition, the proportion of intrinsic subtypes was comparable between control and treated groups with the controls slightly enriched with HER2-E (54% vs 41·4%) and reduced Luminal A tumours (8·0% vs 21·1%), not statistically significantly different (p>0·05), Within the treated subgroup, 31% achieved GR, 22·5% were IRs and 46·5% PRs, while 52·0% had Ki67_2wk_ High and 48·0% Low, respectively.

**Figure 1:**
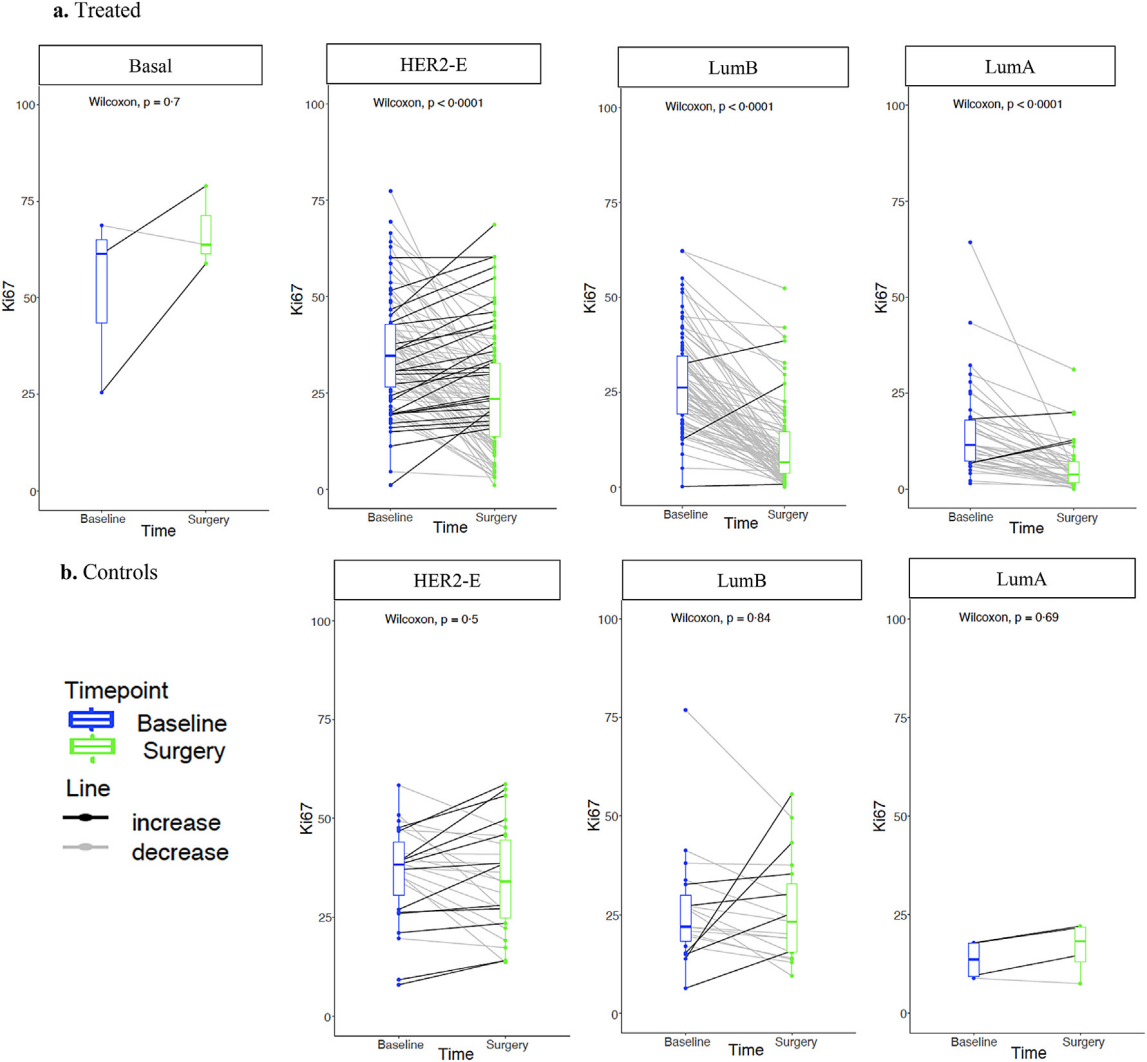
Ki67 changes from baseline to surgery stratified by intrinsic subtype at baseline within a. Treated and b. Controls. Abbreviations: HER2-E, HER2-Enriched Subtype; LumB, Luminal B subtype; LumA, Luminal A subtype.

As expected, most control tumours were classified as PRs (96·0%) and Ki67_2wk_ High (96·0%) (Table 1). In the treated group, there was a significant change in Ki67 values in all subtypes (Figure 1A), except for Basal-like, possibly due to the small sample number. Overall, the HER2-E subtype was associated with poorer response to AI compared to non-HER2-E, evaluated as Ki67 response category and residual Ki67. HER2-E tumours were associated with higher grade (68·6% grade 3 in HER2-E vs 30·9% in luminals, *p*<0·0001, Fisher test) and larger tumours (50·3% >3 cm in HER2-E vs 39·1% in luminals, *p*<0·0001, Fisher test). However, the association of the HER2-E subtype and Ki67 response remained significant in each of the categories within those variables. In the control patients with Ki67 data, no significant changes of Ki67 were observed amongst subtypes (Figure 1B). These findings suggest that HER2-E might be one of the main components driving poor early response to AI in ER+/HER2+ BC tumours.

**Table 1:** Distribution of the 4 intrinsic subtypes within patients with Ki67 data and by Ki67 categories **Abbreviations**: HER2-E, HER2-Enriched Subtype; LumA, Luminal A subtype; LumB, Luminal B subtype; GR, Good response; IR, Intermediate response, PR, Poor response; Ki67_2wk,_ Ki67 at 2 weeks timepoint.

	TREATED					CONTROLS				
Arm	All	Basal	HER2-E	LumA	LumB	All	Basal	HER2-E	LumA	LumB
	226 (100.0%)	3 (1.3%)	95 (42.1%)	45 (19.9%)	83 (36.7%)	50 (100.0%)	0 (0.0%)	27 (54.0%)	4 (8.0%)	19 (38.0%)
Ki67 Response Categories										
**GR**	70 (31·0%)	0 (0·0%)	15 (15·8%)	18 (40·0%)	37 (44·6%)	0 (0·0%)	0 (0·0%)	0 (0·0%)	0 (0·0%)	0 (0·0%)
**IR**	51 (22·5%)	0 (0·0%)	17 (16·5%)	11 (24·4%)	23 (27·7%)	2 (4·0%)	0 (0·0%)	1 (3·80%)	0 (0·0%)	1 (5·3%)
**PR**	105 (46·5%)	3 (100%)	63 (66·3%)	16 (35·5%)	23 (27·8%)	48 (96·0%)	0 (0·0%)	26 (96·3%)	4 (100%)	18 (94·7%)
		Chi -squared 27·69, *P*<0·00001					Fisher's exact test *P*>0·05			
Ki67_2wks_										
**HIGH**	118 (52·0%)	3 (100·0%)	80 (84·2%)	7 (15·6%)	28 (33·7%)	48 (96·0%)	0 (0·0%)	27 (100%)	3 (75·0%)	18 (94·7%)
**LOW**	109 (48·0%)	0 (0·0%)	15 (15·8%)	38 (84·4%)	55 (66·3%)	2 (4·0%)	0 (0·0%)	0 (0·0%)	1 (25·0%)	1 (5·30%)
		Chi-squared 67·98, *P*<0·00001					Fisher's exact test *P*>0·05

### Signature expression and Ki67 endpoints

**Figure 2:**
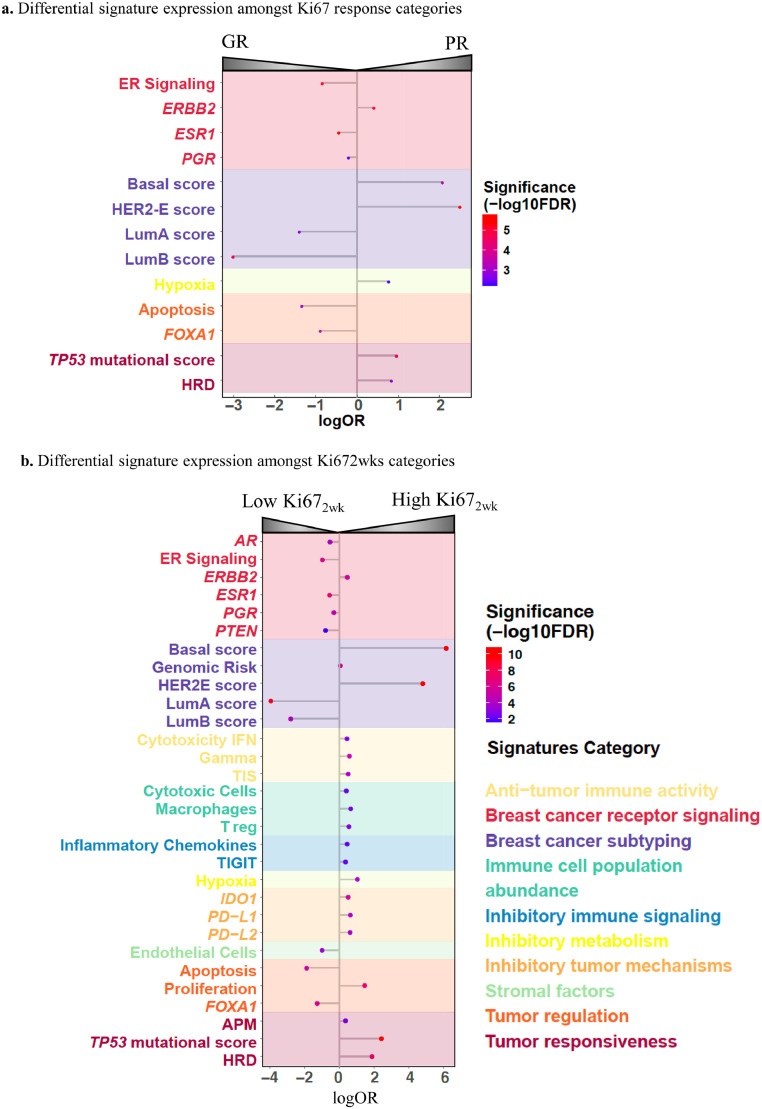
Signatures significantly associated with differential response to AI. a. Ordinal regression models in treated patients with Ki67 data (*n*=227) coloured by FDR for Ki67 response categories and b. Logistic regression models for Ki67_2wk._ Abbreviations: AI, Aromatase inhibitors; logOR, log Odds Ratio; FDR, False discovery rate; HER2-E, HER2-Enriched Subtype; LumB, Luminal B subtype; LumA, Luminal A subtype.

We then evaluated the association of other biological molecular features (46 signatures) with the defined Ki67 endpoints (Supplementary Table S3). High expression of endocrine related signatures such as *ESR1*, ER-Signaling, *FOXA1* and *PgR* as well as Luminal A and B correlation coefficient scores were associated with GR and Low Ki67_2wk_ (OR 0·05–0·82; FDR<0·0001–0·0015, ordinal logistic regression model)_,_ while high *ERBB2*, Basal-like and HER2-E correlation coefficient scores were associated with PR and High Ki67_2wk_ (OR 1·52–12·31; FDR<0·0001, ordinal logistic regression) (Figure 2). Noteworthy, the high expression of apoptosis signature (pro-apoptotic) was associated with GR (OR 0·26; 95% CI 0·12–0·54; FDR=0·0017, ordinal logistic regression) whilst high DNA-damage repair signatures such as the homologous recombination deficiency (HRD), hypoxia, and the *TP53* mutational status’ surrogate signature were associated with PR (OR 2·18–2·65, FDR<0·0001–0·0059, ordinal logistic regression). Additional high expression of signatures involved in immune-checkpoint component and tumour immunity such as *IDO1*, IFN Gamma, *PD-L1* and Tumour Inflammation Signature (TIS) as well as the genomic risk score were associated with High Ki67_2wk_ (OR 1·67–1·49, FDR<0·0001–0·0084, logistic regression). Following prior evidence, we also assessed the correlation of the different signatures with Ki67 > or < than 20% to validate our findings.^17^ The same signatures were associated with differential response to AI based on this Ki67 threshold (Supplementary Table S4).

To assess the above associations according to the different subtypes, we tested them in the HER2-E and Luminal subtypes separately. The signatures were not significantly differently expressed between response categories amongst HER2-E tumours for any of the two Ki67 endpoints (Supplementary Figure S2A). However, in the Luminal tumours, proliferation, *TP53* mutational score, genomic risk and the Basal-like and HER2-E coefficient scores were associated with High Ki67_2wk_ (OR 1·15–16·84; FDR=0·00056–0·013, logistic regression), while high expression of *AR* signature and the Luminal A coefficient scores were associated with Low Ki67_2wk_ (OR 0·25–0·77; FDR=00019–0·013, logistic regression) (Supplementary Figure S2B). Thus, beyond HER2-E intrinsic subtype: *ERBB2, TP53* mutational status, HRD and some immune-related signatures were also associated with early resistance to AI.

### Single gene expression and Ki67 endpoints

**Figure 3:**
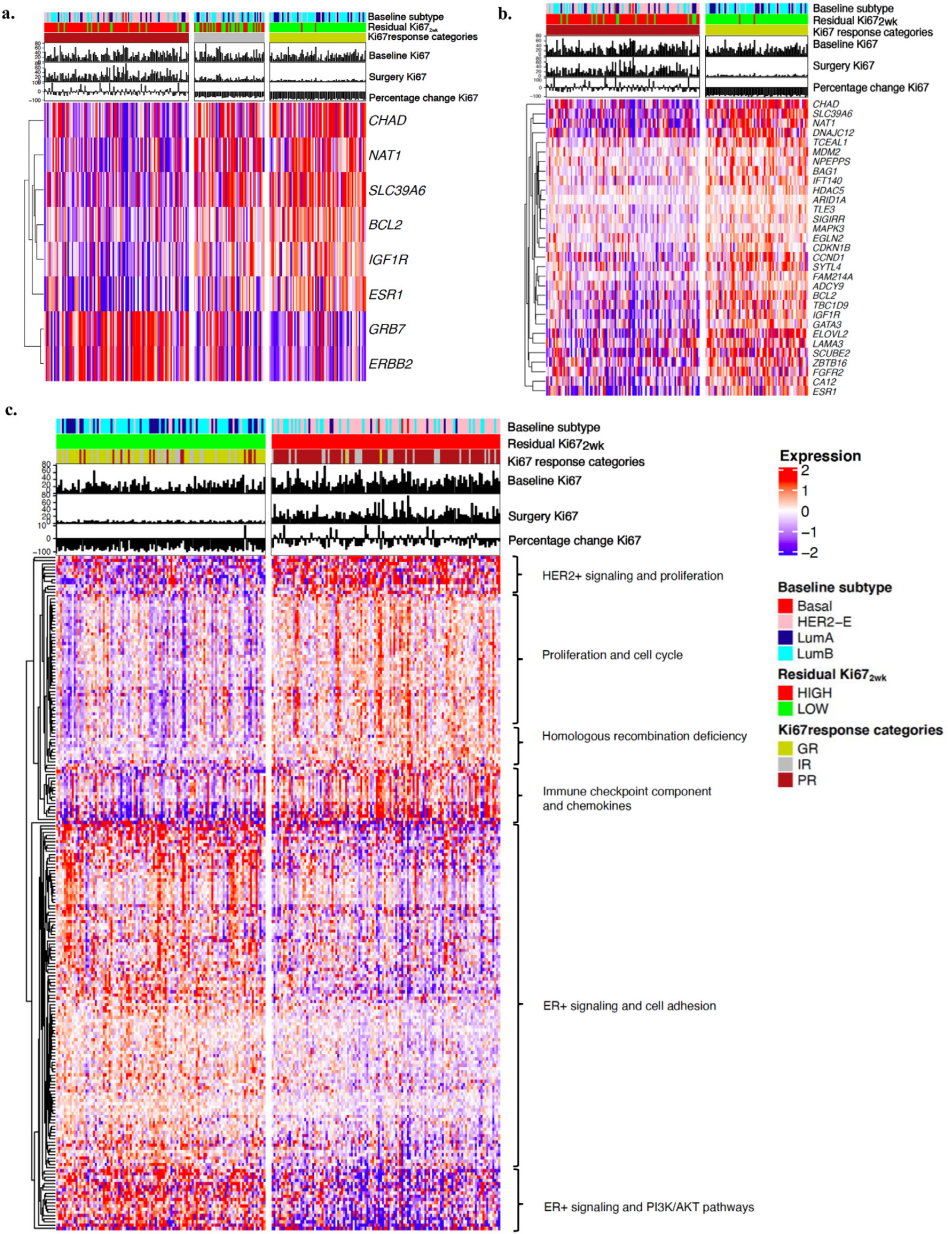
Hierarchical clustering using significant genes from SAM analysis of single gene expression for the different Ki67 endpoints in treated patients. a. Supervised hierarchical clustering of the multiclass SAM analysis for the three Ki67 response categories (GR/IR/PR) (*n*=226). b. Supervised hierarchical clustering of the two unpaired class SAM analysis (*n*=226). c. Supervised hierarchical clustering of the genes selected by two class unpaired SAM analysis for Ki67_2wks_ categories: High vs Low (*n*=227): 211 significant genes, 128 associate with low and 83 with high residual Ki67. Abbreviations: HER2-E, HER2-Enriched Subtype; LumB, Luminal B subtype; LumA, Luminal A subtype; Ki67_2w__k__,_ Ki67 at 2 weeks timepoint.

Multiclass SAM analysis of single gene expression for the three Ki67 response categories (GR/IR/PR) (Δ=0·26, FDR<0·05) identified 8 genes with significantly different expression amongst groups (*p*<0·05). High expression of *GRB7* and *ERBB2* were associated with PR while high expression of others such as *IGF1R, ESR1, CHAD* and *BCL2*, were associated with GR (Figure 3A). Two unpaired class SAM analysis for GR versus PR (Δ=1, FDR<0·05) showed the association of 31 genes with GR (*p* <0·05, Wilcoxon test) (Figure 3B)· Two class unpaired SAM analysis with Ki67_2wk_ categories: High versus Low (Δ=1·63, median FDR=0) identified 128 genes associated to Low Ki67_2wk_ including genes involved in *PI3K/AKT, MAPK* and oestrogen signaling and 83 genes associated to High Ki67_2wk,_ including genes involved in immune-checkpoint component, proliferation and cell-cycle regulation (Figure 3C).

To further investigate the biological differences between early AI responders and resistant tumours we evaluated the *ESR1* gene expression levels amongst the four intrinsic subtypes. The highest levels of *ESR1* were seen in Luminal B tumours. Noteworthy, within the Luminal A tumours a group of patients showed lower levels of *ESR1* were associated with higher levels of *ERBB2* signaling, especially when compared with other subtypes (Supplementary Figure S3).

Overall, at a single gene expression level, ER signalling-related genes drive responses to AI treatment while *HER2* and immune-related genes associates with early resistance.

### Identification of new molecular subgroups predicting Ki67 endpoints

**Figure 4:**
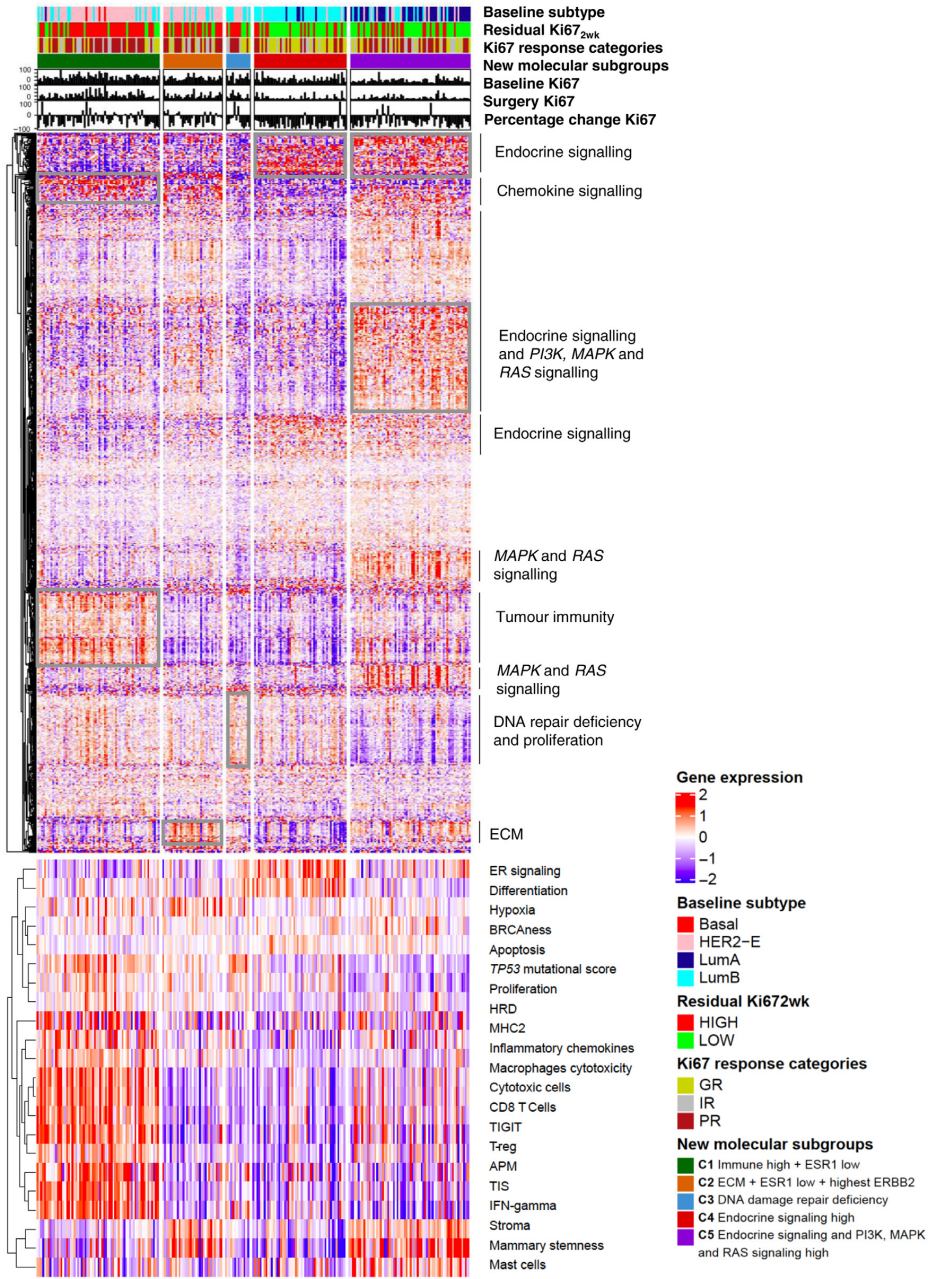
Five new molecular subgroups were identified based on consensus clustering of tumours using expression of 758 genes. Top panel illustrates single gene expression of all genes in treated patients with Ki67 data only (*n*=226), separated by the 5 novel molecular subgroups. Clusters of genes are annotated according to their functionality. Bottom panel illustrates the expression levels of the 21 multigene signatures, ordered according to the new 5 molecular subgroups. Abbreviations: HER2-E, HER2-Enriched Subtype; LumB, Luminal B subtype; LumA, Luminal A subtype; GR, Good responders; IR, Intermediate responders, PR, Poor Responders; Ki67_2wk,_ Ki67 at 2 weeks timepoint, ER, Oestrogen receptor; HRD, Homologous recombination deficiency ECM, extracellular matrix; TIS, Tumour Inflammation Signature; TIGIT, T cell immunoreceptor with Ig and ITIM domain; MHC, Major Histocompatibility complex APM, Antigen Processing Machinery, IFN-gamma, interferon gamma.

Using consensus clustering, we identified 5 novel molecular subgroups of samples based on single-gene expression that are associated with different Ki67 response and Ki67_2wk_ (Figure 4 and Supplementary Table S5). Interestingly, these new molecular subgroups divided mainly HER2-E samples with lower response to AI, into three groups with differential expression of molecular features such as *ERBB2*, ER signaling or immune-related pathways.

**Figure 5:**
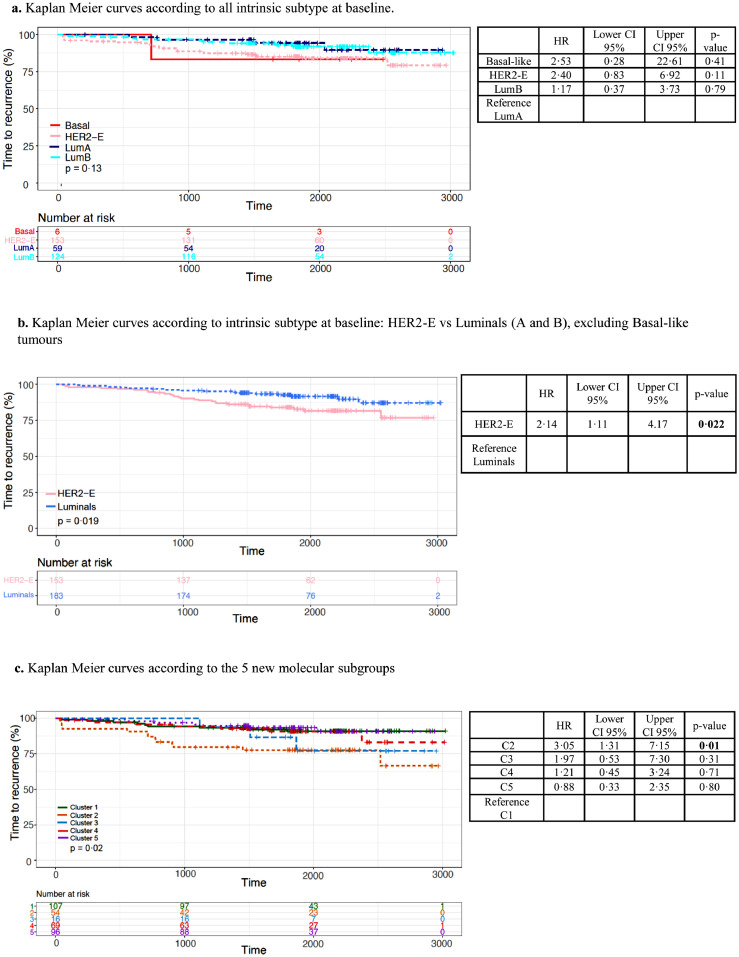
Kaplan Meier curves for TTR according to a. all PAM50 intrinsic subtype at baseline; b. HER2-E vs luminals and c. the new molecular subgroups. Abbreviations: TTR: Time to recurrence; HER2-E, HER2-enriched; LumB; Luminal B.

Figure 4 shows the molecular features of the clusters (C): C1, C2 and C3 were characterised by PR to AI (60/111, 54·1%) and high Ki67_2wk_ (82/111, 73·9%) and an enrichment of HER2-E subtype (79/111; 71·2%) and Luminal B tumours (21/111, 18·9%) (Supplementary Figure S4A). C1 (29·2% of the total) showed higher levels of immune and chemokine-related genes and lower levels of *ESR1* (Supplementary Figure S4B). C2 (14·2% of the total) had higher levels of extracellular matrix organization (ECM) related genes, lower levels of *ESR1* and the highest levels of *ERBB2* (Supplementary Figure S4C), also observed when distributed by subtypes. C3 (5.7% of the total) had low expression of most genes but a slight upregulation of genes covering DNA-damage repair deficiency*.* C4 (22.1%) and C5 (28·8%) were characterised by GR to AI and an enrichment of luminal tumours (Luminal B 81/165; 49·1% and Luminal A 57/165; 34·5%) (Supplementary Figure S4A and Supplementary Table S5). Both luminal subgroups overexpressed ER signaling-related genes. However, while C4 had the highest levels of *ESR1* (Figure 5C), C5 was enriched with genes involved in *MAPK/PI3K* and *RAS* signaling. Figure 4 also shows the signatures’ expression within each of the single gene-based molecular subgroups, thus confirming the biologic distinctiveness of each of the new molecular subgroups.

### Time to recurrence analysis

Median follow-up was 62·9 months (IQR 58·1–74·1). Univariate analysis was performed to evaluate the prognostic value of each individual genomic feature analysed. There was no overall significant difference of TTR between intrinsic subtypes when all the subgroups were compared together (Figure 5A). However, HER2-E had significantly poorer TTR (HR 2·14; 95% CI 1·11–4·17; *p*=0·022, univariate regression model) compared to luminal tumours (Figure 5B). C2, which is characterised by low expression of immune-related genes and the highest *ERBB2* expression, showed a significantly higher risk of recurrence compared to the rest of the molecular subgroups (HR 3·05; 95% CI 1·31–7·15; *p*=0·01, univariate regression model) (Figure 5C).

We performed a series of cox regression models for multivariable survival analysis including standard clinicopathologic factors along with different molecular subgroups for TTR and compared the changes of chi-squared values between them to assess the added value of the different models (Supplementary Table S6). HER2-E subtype remained as an independent predictor of higher risk of relapse (HR 2.55, 95% CI 1·14–5·69; *p*=0·022, multivariable regression model). C2 was also an independent predictor of shorter TTR compared to C1, indicating that this model adds significant value beyond intrinsic subtypes (Likelihood ratio test, *p*= 0.0025). (Supplementary Figure S5).

To explore further the role of adjuvant HER2-targeted treatment and chemotherapy on survival, we replicated the same analysis but separately for those patients that had received both of these treatments and those that did not (Supplementary Figure S6). Noteworthy, among patients who had received adjuvant trastuzumab plus chemotherapy, there were no significant differences in TTR observed between HER2-E and other subtypes (Supplementary Figure S6a). However, among patients who had not received trastuzumab and chemotherapy, HER2-E was still significantly associated with worse outcome compared to non-HER2-E (Supplementary Figure S6a). For the new molecular subgroups, the survival outcome within the new molecular subgroup 2 was worse compared to the others in those patients receiving adjuvant anti-HER2 treatment. In addition, in the multivariable models, the new molecular subgroups remained as independent predictors of TTR restricting to patients who received trastuzumab plus chemotherapy (HR 3.95; 95% CI 1·04–14·94; *p*=0·0043, multivariate regression model).

Finally, a series of multivariable analyses for TTR of signature expression adjusted for the clinicopathological factors showed that several immune related signatures (HR 0.50–0·78, nominal *p* <0·0001–0·0430, multivariable regression model) as well as the apoptosis signature (HR 0·36, 95% CI 0·15–0·38; *p*=0·024, multivariable regression model) were independent predictors of better TTR. By contrast, claudin-low signature showed independent worse prognostic value (HR 1.26, 95% CI 1·06–1·56; *p*=0·031, multivariable regression model) (Supplementary Table S7). A final model including all identified features was not performed due to the high collinearity amongst the main significant signatures (Supplementary Figure S7).

## Discussion

AI treatment is the standard of care and most effective therapy for post-menopausal women with early ER+ BC. However, ER+ tumours that also over-express HER2, show limited response to ET and thus, are at a higher risk of recurrence.^12^ Most studies performed in ER+/HER2+ BC have focused on resistance to anti-HER2 targeted therapy while mechanisms of resistance to ET are not well understood within this subgroup. This study investigated the predictive and prognostic value of molecular features at baseline in ER+/HER2+ BC treated with perioperative AI.

Prior studies have revealed that HER2-E subtype is a predictor of higher sensitivity to anti-HER2 targeted therapy but worse outcome than other subtypes such as luminal tumours.^5^^,^^17^, ^18^, ^19^ Nevertheless, the role of the intrinsic subtypes in response to ET has not been well established yet. A prior single-arm, multicentric study (PerELISA) included 65 postmenopausal patients with HR+/HER2+ operable patients receiving 2 weeks of letrozole and then undergoing to re-biopsy for Ki67 evaluation.^17^ This study reported the association of PAM50 intrinsic subtyping with molecular responders (Ki67 relative reduction >20% from baseline). 92% of responders were luminal A and B versus 44% HER2-E and basal-like (*P* < 0·001). These results were aligned with ours; however, this is a small, single arm, non-randomised study, while POETIC was a randomised trial, enabling us to evaluate the predictive value of the intrinsic subtyping). Within PerELISA trial patients classified as molecular responders continued letrozole and started trastuzumab-pertuzumab for five cycles. They also reported that the pCR rate was significantly higher in HER2-E than in other subtypes (45·5% versus 13·8%, P=0·042). There was no association test of survival outcome reported, nevertheless, these results are of clinical significance as this provide a potential strategy for de-escaling the treatment management

Our results identified HER2-E subtype as a predictive biomarker of resistance to AI in ER+/HER2+ BC with an additional higher risk of relapse.^20^^,^^21^ These findings highlight two aspects. First, they suggest the potential need of treatment intensification for HER2-E ER+/HER2+ BC tumours with intensive anti-HER2 targeted therapy. Second, the higher sensitivity to AI and good prognosis associated with luminal tumours, in particular with Luminal A, provides a rationale for testing de-escalation approaches, such as the reduction of anti-HER2 blockade duration or avoiding chemotherapy, previously suggested.^22^ A related finding was published in 2021 as part of the ADAPT HER2+ trial in which the pooled TDM1 arm of patients with HER2-E subtype had a higher pCR, which was not observed in the trastuzumab arm.^23^

Nowadays, the biggest question is how to select patients for escalation and de-escalation strategies. Recent “chemotherapy-free” neoadjuvant studies have shown that the combination of HER2-E subtype with high *ERBB2* mRNA expression may identify patients with HER2+ early BC with higher sensitivity to double HER2-blockade.^18^ However, only one third of ER+/HER+ BC patients are HER2-E/*ERBB2* high and their role as prognostic biomarkers is still unknown. Using consensus clustering, we identified new molecular subgroups based on single gene expression at a higher risk of relapse beyond HER2-E subtype. Results from the stratified analysis by adjuvant treatment (chemotherapy plus anti-HER2 treatment yes vs no) showed similar TTR despite intrinsic subtype in those patients receiving trastuzumab plus chemotherapy. This highlights the hypothesised notion that patients with HER2-E subtype benefit the most from anti-HER2 treatment. The independent prognostic value for the new molecular subgroups for TTR in patients who received trastuzumab plus chemotherapy suggests the potential clinical utility of these new molecular subgroups to identify a set of ER+/HER2+ patients, beyond intrinsic subtypes, who may benefit from emerging treatment such as CDK4/6 inhibitors.

Noteworthy, tumours characterised by an enrichment of immune features showed the lowest risk of recurrence despite immune features being predictive markers of resistance to AI and poor outcome in ER+/HER2- BC.^24^^,^^25^ Due to its intrinsic good prognosis, BC patients within this molecular subgroup showing an enrichment of immune characteristics could potentially benefit the most from a de-escalating approach, using for example 6 month of trastuzumab plus AI without chemotherapy followed by 5 years of AI treatment alone. Several trials in HER2+ BC have shown that tumours with higher baseline tumour infiltrating lymphocytes (TILs) and other immune features achieve higher pathological complete response rates and improved event-free survival.^26^ Our study confirms the association of higher expression of immune-features with a very low risk of recurrence in ER+/HER2+ BC. By contrast, tumours with higher levels of *ERBB2* and lower associated immunity had a significantly higher risk of relapse, indicating that these patients may benefit from an intensified anti-HER2 treatment, using for example double anti-HER2 blockade or adjuvant TDM1.^27^^,^^28^ Therefore, these new molecular subgroups might be essential to identify candidates for new escalating and de-escalating strategies.

The role of tumour immunity in response to ET has been mainly studied in ER+/HER2- BC disease, with higher expression of genes involved in immune enrichment and targetable immune checkpoint components being correlated with higher risk such as Luminal B tumours.^24^^,^^25^ Our study shows that higher tumour immunity might also be a key driver of early resistance to ET in ER+/HER2+ BC. However, in this subgroup the association of higher expression of immune-features and lower risk of recurrence indicates the different role of tumour immunity between HER2+ and HER2- disease and may suggest a de-escalation approach.

Furthermore, our study shows other striking molecular associations such as high expression of HRD and *TP53* signature predicting poor response to AI, and higher apoptosis signaling being associated with good response and survival. The association of DNA damage repair defects with resistance to ET has been previously reported.^29^ In addition, the inhibition of poly (ADP-ribose) polymerase-1 (PARP), has shown anti-tumour effects with a strong synergism and good tolerance in combination with anti-HER2 targeted therapy or ET *in vitro* and in phase I/II trials independently of DNA repair deficiency.^30^ *TP53* mutational status has also been previously linked with poor survival^31^ and overexpression of *HER2,*^32^ although its role in response to AI is still unclear. Previous data has shown that some molecular features related to apoptosis can be predictive of adjuvant benefit from ET.^33^ Based on our results, the association of high expression of some of the signatures reported above such as immune-related signatures and *TP53* mutational score with poor AI response could be explained by the high correlation with the HER2-E subtype.

Our study has two main limitations. Firstly, we only analysed gene expression for 758 genes and intrinsic subtypes profiles using the BC360 platform and other important pathways may have been missed. Secondly, clinical practice is currently different to that implemented in the recruited POETIC patients as high-risk tumours would be receiving additional pertuzumab and further anti-HER2 agents such as TDM1. It is noteworthy that one third of the patients in our cohort did not receive any adjuvant treatment apart from ET due to their advanced age, but we adjusted the survival analysis for age as a main surrogate of treatment choice. The major strengths of our study are that to our knowledge this is the largest cohort investigating response to pre-surgical AI in ER+/HER2+ subgroup in a real-world cohort which has a unique value to assess global gene expression data at baseline as defined in the clinical practice. Lastly, we were able to analyse both the molecular features associated to mechanisms of resistance and their prognostic value.

In conclusion, HER2-E subtype and *ERBB2* play a crucial role in ER+/HER2+ BC, driving resistance to ET and a higher risk of recurrence. Beyond them, new molecular subgroups enable the identification of patients at a higher risk of relapse. Altogether, the combination of these biomarkers would lead to a better tailoring of treatment strategies, including escalation and de-escalation approaches, to improve resistance to treatment in early BC. The appropriate new strategies need to be addressed in prospective clinical trials.

## Contributors

MB and ELK designed, conducted part of the research work, analysed and verified the data and wrote the original draft of the manuscript. GM conducted part of the research work. HT did data analysis and interpretation. LK participated in study design, data collection and data interpretation. ES supervised some of the analysis of the data. AA and MH conducted part of the research work. HX participated in data analysis for manuscript revision. CH recruited patients to the POETIC trial and provided tissue. AS participated in POETIC study design, POETIC trial management group and clinical recruitment. JFR participated in POETIC trial design and reviewing the manuscript. IS participated in the design of original POETIC trial and was chief investigator of it. JMB has provided oversight and guidance for trial management, statistics and data interpretation throughout the trial including insights into the translational research project presented here. She reviewed and contributed to the manuscript. MD was involved in the investigation and visualisation of the study. MCUC designed and led the study. She also led the conceptualisation, methodology, resources and funding acquisition, data curation and statistical analysis plan, supervision. All authors made substantial contributions to the manuscript, revised the manuscript critically, gave their final approval and had full access to all the data and accept responsibility to submit for publication.

## Data sharing statement

De-identified data will be made available to other researchers on request, subject to approval of a formal data access request in accordance with the ICR-CTSU data and sample access policy. Gene expression data is available on request by contacting poetic-icrctsu@icr.ac.uk. The ICR-CTSU supports the wider dissemination of information from the research it does, and increased cooperation between investigators. Trial data is collected, managed, stored, shared, and archived according to ICR-CTSU Standard Operating Procedures in order to ensure the enduring quality, integrity, and utility of the data. Formal requests for data sharing are considered in line with the Institute of Cancer Research Clinical Trials and Statistics Unit (ICR-CTSU) procedures with due regard given to funder and sponsor guidelines. Requests are via a standard proforma describing the nature of the proposed research and extent of data requirements. Data recipients are required to enter a formal data sharing agreement which describes the conditions for release and requirements for data transfer, storage, archiving, publication and intellectual property. Requests are reviewed by the Trial Management Group (TMG) in terms of scientific merit and ethical considerations including patient consent. Data sharing is allowed if proposed projects have a sound scientific or patient benefit rationale as agreed by the TMG and approved by the Trial Steering Committee as required. Restrictions relating to patient confidentiality and consent will be limited by aggregating and anonymising identifiable patient data.

## Declaration of interests

MB reports grants from Fundacion Alonso Martin Escudero and has a patent filed for the novel molecular subgroups identified in this paper. ELK has a patent filed for the novel molecular subgroups identified in this paper. HT and LK report grants from Cancer Research UK, during the conduct of the study. JMB reports grants from Cancer Research UK, during the conduct of the study; grants and non-financial support from AstraZeneca, Merck Sharp & Dohme, Puma Biotechnology, Clovis Oncology, Pfizer, Janssen-Cilag, Novartis, Eli Lilly, and Roche, outside the submitted work. MD receives consulting fees from AstraZeneca, Lilly, Roche, Radius, H3 Biomedicine and G1. He also receives honoraria from Nanostring. M.C.U.C. has a patent for Breast Cancer Classifier: US Patent No. 9,631,239 with royalties paid and receive research funding from NanoString Technologies and Veracyte advisory role. And has a patent filed for the novel molecular subgroups identified in this paper. All other authors declare no competing interests. The information in this manuscript is subject to a pending patent application.

## Appendix Supplementary materials

Image, application 1

Image, application 2

Image, application 3

## References

[1] Slamon DJ, Clark GM, Wong SG (1987). Human breast cancer: correlation of relapse and survival with amplification of the HER-2/neu oncogene. Science.

[2] Perez EA, Romond EH, Suman VJ (2014). Trastuzumab plus adjuvant chemotherapy for human epidermal growth factor receptor 2-positive breast cancer: planned joint analysis of overall survival from NSABP B-31 and NCCTG N9831. J Clin Oncol.

[3] Early Breast Cancer Trialists' Collaborative g (2021). Trastuzumab for early-stage, HER2-positive breast cancer: a meta-analysis of 13 864 women in seven randomised trials. Lancet Oncol.

[4] Cameron D, Piccart-Gebhart MJ, Gelber RD (2017). 11 years' follow-up of trastuzumab after adjuvant chemotherapy in HER2-positive early breast cancer: final analysis of the HERceptin Adjuvant (HERA) trial. Lancet.

[5] Cejalvo JM, Pascual T, Fernandez-Martinez A (2018). Clinical implications of the non-luminal intrinsic subtypes in hormone receptor-positive breast cancer. Cancer Treat Rev.

[6] Prat A, Perou CM. (2011). Deconstructing the molecular portraits of breast cancer. Mol Oncol.

[7] Prat A, Baselga J. (2008). The role of hormonal therapy in the management of hormonal-receptor-positive breast cancer with co-expression of HER2. Nat Clin Pract Oncol.

[8] Martin LA, Ribas R, Simigdala N (2017). Discovery of naturally occurring ESR1 mutations in breast cancer cell lines modelling endocrine resistance. Nat Commun.

[9] Lopez-Knowles E, Pearson A, Schuster G (2019). Molecular characterisation of aromatase inhibitor-resistant advanced breast cancer: the phenotypic effect of ESR1 mutations. Br J Cancer.

[10] Smith I, Robertson J, Kilburn L (2020). Long-term outcome and prognostic value of Ki67 after perioperative endocrine therapy in postmenopausal women with hormone-sensitive early breast cancer (POETIC): an open-label, multicentre, parallel-group, randomised, phase 3 trial. Lancet Oncol.

[11] Pinhel IF, Macneill FA, Hills MJ (2010). Extreme loss of immunoreactive p-Akt and p-Erk1/2 during routine fixation of primary breast cancer. Breast Cancer Res.

[12] Dowsett M, Ebbs SR, Dixon JM (2005). Biomarker changes during neoadjuvant anastrozole, tamoxifen, or the combination: influence of hormonal status and HER-2 in breast cancer–a study from the IMPACT trialists. J Clin Oncol.

[13] Benjamini Y, Hochberg Y. (1995). Controlling the false discovery rate: a practical and powerful approach to multiple testing. J R Stat Soc Ser B (Methodol).

[14] Tusher VG, Tibshirani R, Chu G. (2001). Significance analysis of microarrays applied to the ionizing radiation response. Proc Natl Acad Sci USA.

[15] Gu Z, Eils R, Schlesner M. (2016). Complex heatmaps reveal patterns and correlations in multidimensional genomic data. Bioinformatics.

[16] Monti S, Tamayo P, Mesirov J, Golub T. (2003). Consensus clustering: a resampling-based method for class discovery and visualization of gene expression microarray data. Mach Learn.

[17] Guarneri V, Dieci MV, Bisagni G (2019). De-escalated therapy for HR+/HER2+ breast cancer patients with Ki67 response after 2-week letrozole: results of the PerELISA neoadjuvant study. Ann Oncol.

[18] Prat A, Pascual T, De Angelis C (2020). HER2-enriched subtype and ERBB2 expression in HER2-positive breast cancer treated with dual HER2 blockade. J Natl Cancer Inst.

[19] Schettini F, Pascual T, Conte B (2020). HER2-enriched subtype and pathological complete response in HER2-positive breast cancer: a systematic review and meta-analysis. Cancer Treat Rev.

[20] Perou CM, Sorlie T, Eisen MB (2000). Molecular portraits of human breast tumours. Nature.

[21] Cancer Genome Atlas N (2012). Comprehensive molecular portraits of human breast tumours. Nature.

[22] File D, Curigliano G, Carey LA. (2020). Escalating and de-escalating therapy for early-stage HER2-positive breast cancer. Am Soc Clin Oncol Educ Book.

[23] Harbeck N, von Schumann R, Kates RE (2021). Immune markers and tumor-related processes predict neoadjuvant therapy response in the WSG-ADAPT HER2-positive/hormone receptor-positive trial in early breast cancer. Cancers (Basel).

[24] Dunbier AK, Ghazoui Z, Anderson H (2013). Molecular profiling of aromatase inhibitor-treated postmenopausal breast tumors identifies immune-related correlates of resistance. Clin Cancer Res.

[25] Anurag M, Zhu M, Huang C (2020). Immune checkpoint profiles in luminal B breast cancer (Alliance). J Natl Cancer Inst.

[26] Salgado R, Denkert C, Campbell C (2015). Tumor-infiltrating lymphocytes and associations with pathological complete response and event-free survival in HER2-positive early-stage breast cancer treated with lapatinib and trastuzumab: a secondary analysis of the NeoALTTO trial. JAMA Oncol.

[27] von Minckwitz G, Huang C-S, Mano MS (2018). Trastuzumab emtansine for residual invasive HER2-positive breast cancer. N Engl J Med.

[28] von Minckwitz G, Procter M, de Azambuja E (2017). Adjuvant pertuzumab and trastuzumab in early HER2-positive breast cancer. N Engl J Med.

[29] Anurag M, Punturi N, Hoog J (2018). Comprehensive profiling of DNA repair defects in breast cancer identifies a novel class of endocrine therapy resistance drivers. Clin Cancer Res.

[30] Keung MY, Wu Y, Badar F, Vadgama JV. (2020). Response of breast cancer cells to PARP inhibitors is independent of BRCA status. J Clin Med.

[31] Baker L, Quinlan PR, Patten N (2010). p53 mutation, deprivation and poor prognosis in primary breast cancer. Br J Cancer.

[32] Roman-Rosales AA, Garcia-Villa E, Herrera LA, Gariglio P, Diaz-Chavez J. (2018). Mutant p53 gain of function induces HER2 over-expression in cancer cells. BMC Cancer.

[33] Dowsett M, Smith IE, Ebbs SR (2006). Proliferation and apoptosis as markers of benefit in neoadjuvant endocrine therapy of breast cancer. Clin Cancer Res.

