# Supplementary material for: HER2-enriched subtype and novel molecular subgroups drive aromatase inhibitor resistance and an increased risk of relapse in early ER+/HER2+ breast cancer
Source: eBioMedicine. 2022 Aug 16;83:104205. doi: 10.1016/j.ebiom.2022.104205 (PMC9482930; doi:10.1016/j.ebiom.2022.104205)
Supplement: Supplementary file 1 [file mmc1.pptx]

## Slide 1
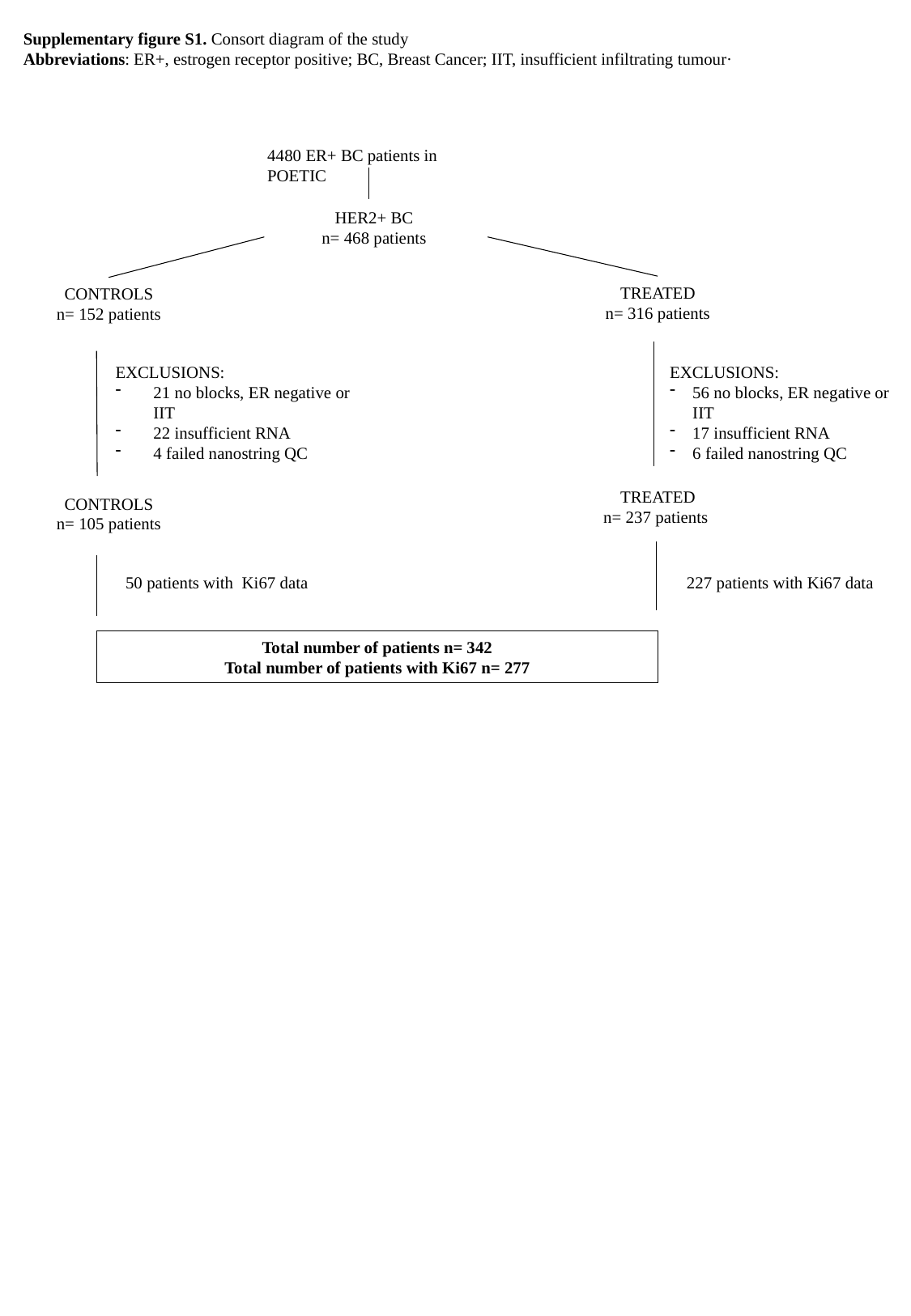

Supplementary figure S1. Consort diagram of the study
Abbreviations: ER+, estrogen receptor positive; BC, Breast Cancer; IIT, insufficient infiltrating tumour·
4480 ER+ BC patients in POETIC
HER2+ BC
n= 468 patients
TREATED
n= 316 patients
CONTROLS
n= 152 patients
EXCLUSIONS:
21 no blocks, ER negative or IIT
22 insufficient RNA
4 failed nanostring QC
EXCLUSIONS:
56 no blocks, ER negative or IIT
17 insufficient RNA
6 failed nanostring QC
TREATED
n= 237 patients
CONTROLS
n= 105 patients
50 patients with Ki67 data
227 patients with Ki67 data
Total number of patients n= 342
Total number of patients with Ki67 n= 277

## Slide 2
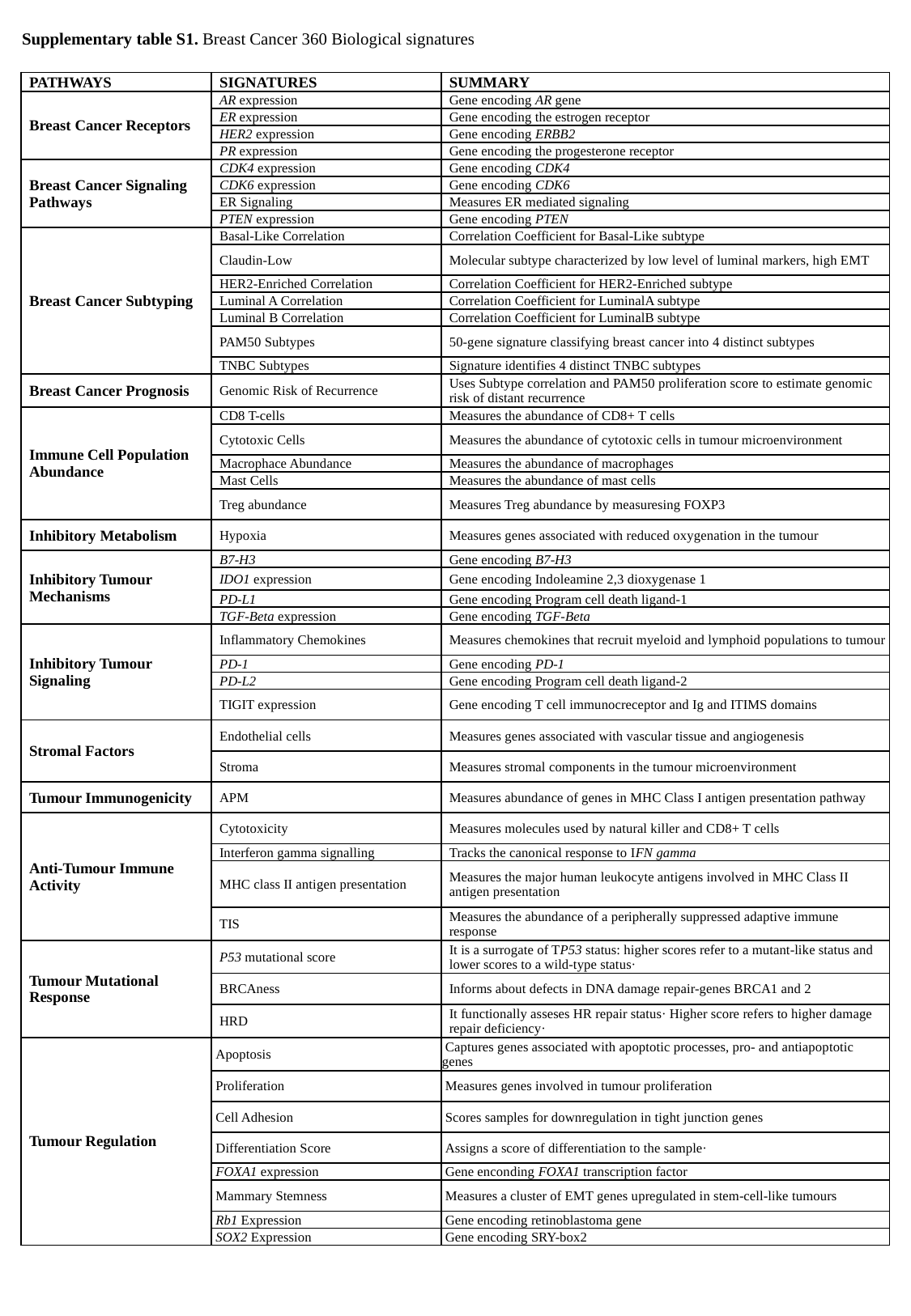

Supplementary table S1. Breast Cancer 360 Biological signatures
| PATHWAYS | SIGNATURES | SUMMARY |
| --- | --- | --- |
| Breast Cancer Receptors | AR expression | Gene encoding AR gene |
| | ER expression | Gene encoding the estrogen receptor |
| | HER2 expression | Gene encoding ERBB2 |
| | PR expression | Gene encoding the progesterone receptor |
| Breast Cancer Signaling Pathways | CDK4 expression | Gene encoding CDK4 |
| | CDK6 expression | Gene encoding CDK6 |
| | ER Signaling | Measures ER mediated signaling |
| | PTEN expression | Gene encoding PTEN |
| Breast Cancer Subtyping | Basal-Like Correlation | Correlation Coefficient for Basal-Like subtype |
| | Claudin-Low | Molecular subtype characterized by low level of luminal markers, high EMT |
| | HER2-Enriched Correlation | Correlation Coefficient for HER2-Enriched subtype |
| | Luminal A Correlation | Correlation Coefficient for LuminalA subtype |
| | Luminal B Correlation | Correlation Coefficient for LuminalB subtype |
| | PAM50 Subtypes | 50-gene signature classifying breast cancer into 4 distinct subtypes |
| | TNBC Subtypes | Signature identifies 4 distinct TNBC subtypes |
| Breast Cancer Prognosis | Genomic Risk of Recurrence | Uses Subtype correlation and PAM50 proliferation score to estimate genomic risk of distant recurrence |
| Immune Cell Population Abundance | CD8 T-cells | Measures the abundance of CD8+ T cells |
| | Cytotoxic Cells | Measures the abundance of cytotoxic cells in tumour microenvironment |
| | Macrophace Abundance | Measures the abundance of macrophages |
| | Mast Cells | Measures the abundance of mast cells |
| | Treg abundance | Measures Treg abundance by measuresing FOXP3 |
| Inhibitory Metabolism | Hypoxia | Measures genes associated with reduced oxygenation in the tumour |
| Inhibitory Tumour Mechanisms | B7-H3 | Gene encoding B7-H3 |
| | IDO1 expression | Gene encoding Indoleamine 2,3 dioxygenase 1 |
| | PD-L1 | Gene encoding Program cell death ligand-1 |
| | TGF-Beta expression | Gene encoding TGF-Beta |
| Inhibitory Tumour Signaling | Inflammatory Chemokines | Measures chemokines that recruit myeloid and lymphoid populations to tumour |
| | PD-1 | Gene encoding PD-1 |
| | PD-L2 | Gene encoding Program cell death ligand-2 |
| | TIGIT expression | Gene encoding T cell immunocreceptor and Ig and ITIMS domains |
| Stromal Factors | Endothelial cells | Measures genes associated with vascular tissue and angiogenesis |
| | Stroma | Measures stromal components in the tumour microenvironment |
| Tumour Immunogenicity | APM | Measures abundance of genes in MHC Class I antigen presentation pathway |
| Anti-Tumour Immune Activity | Cytotoxicity | Measures molecules used by natural killer and CD8+ T cells |
| | Interferon gamma signalling | Tracks the canonical response to IFN gamma |
| | MHC class II antigen presentation | Measures the major human leukocyte antigens involved in MHC Class II antigen presentation |
| | TIS | Measures the abundance of a peripherally suppressed adaptive immune response |
| Tumour Mutational Response | P53 mutational score | It is a surrogate of TP53 status: higher scores refer to a mutant-like status and lower scores to a wild-type status· |
| | BRCAness | Informs about defects in DNA damage repair-genes BRCA1 and 2 |
| | HRD | It functionally asseses HR repair status· Higher score refers to higher damage repair deficiency· |
| Tumour Regulation | Apoptosis | Captures genes associated with apoptotic processes, pro- and antiapoptotic genes |
| | Proliferation | Measures genes involved in tumour proliferation |
| | Cell Adhesion | Scores samples for downregulation in tight junction genes |
| | Differentiation Score | Assigns a score of differentiation to the sample· |
| | FOXA1 expression | Gene enconding FOXA1 transcription factor |
| | Mammary Stemness | Measures a cluster of EMT genes upregulated in stem-cell-like tumours |
| | Rb1 Expression | Gene encoding retinoblastoma gene |
| | SOX2 Expression | Gene encoding SRY-box2 |

## Slide 3
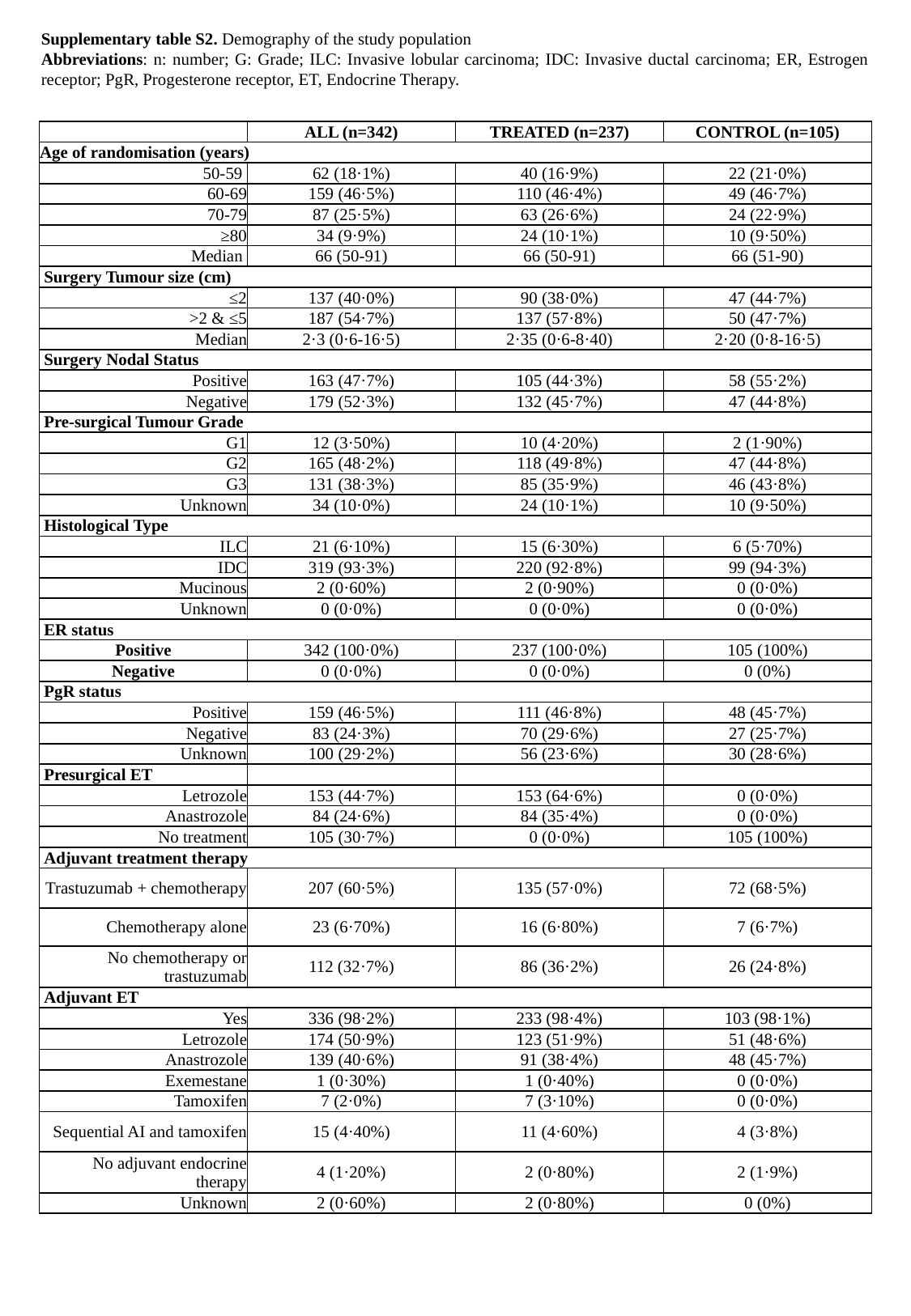

Supplementary table S2. Demography of the study population
Abbreviations: n: number; G: Grade; ILC: Invasive lobular carcinoma; IDC: Invasive ductal carcinoma; ER, Estrogen receptor; PgR, Progesterone receptor, ET, Endocrine Therapy.
| | ALL (n=342) | TREATED (n=237) | CONTROL (n=105) |
| --- | --- | --- | --- |
| Age of randomisation (years) | | | |
| 50-59 | 62 (18·1%) | 40 (16·9%) | 22 (21·0%) |
| 60-69 | 159 (46·5%) | 110 (46·4%) | 49 (46·7%) |
| 70-79 | 87 (25·5%) | 63 (26·6%) | 24 (22·9%) |
| ≥80 | 34 (9·9%) | 24 (10·1%) | 10 (9·50%) |
| Median | 66 (50-91) | 66 (50-91) | 66 (51-90) |
| Surgery Tumour size (cm) | | | |
| ≤2 | 137 (40·0%) | 90 (38·0%) | 47 (44·7%) |
| >2 & ≤5 | 187 (54·7%) | 137 (57·8%) | 50 (47·7%) |
| Median | 2·3 (0·6-16·5) | 2·35 (0·6-8·40) | 2·20 (0·8-16·5) |
| Surgery Nodal Status | | | |
| Positive | 163 (47·7%) | 105 (44·3%) | 58 (55·2%) |
| Negative | 179 (52·3%) | 132 (45·7%) | 47 (44·8%) |
| Pre-surgical Tumour Grade | | | |
| G1 | 12 (3·50%) | 10 (4·20%) | 2 (1·90%) |
| G2 | 165 (48·2%) | 118 (49·8%) | 47 (44·8%) |
| G3 | 131 (38·3%) | 85 (35·9%) | 46 (43·8%) |
| Unknown | 34 (10·0%) | 24 (10·1%) | 10 (9·50%) |
| Histological Type | | | |
| ILC | 21 (6·10%) | 15 (6·30%) | 6 (5·70%) |
| IDC | 319 (93·3%) | 220 (92·8%) | 99 (94·3%) |
| Mucinous | 2 (0·60%) | 2 (0·90%) | 0 (0·0%) |
| Unknown | 0 (0·0%) | 0 (0·0%) | 0 (0·0%) |
| ER status | | | |
| Positive | 342 (100·0%) | 237 (100·0%) | 105 (100%) |
| Negative | 0 (0·0%) | 0 (0·0%) | 0 (0%) |
| PgR status | | | |
| Positive | 159 (46·5%) | 111 (46·8%) | 48 (45·7%) |
| Negative | 83 (24·3%) | 70 (29·6%) | 27 (25·7%) |
| Unknown | 100 (29·2%) | 56 (23·6%) | 30 (28·6%) |
| Presurgical ET | | | |
| Letrozole | 153 (44·7%) | 153 (64·6%) | 0 (0·0%) |
| Anastrozole | 84 (24·6%) | 84 (35·4%) | 0 (0·0%) |
| No treatment | 105 (30·7%) | 0 (0·0%) | 105 (100%) |
| Adjuvant treatment therapy | | | |
| Trastuzumab + chemotherapy | 207 (60·5%) | 135 (57·0%) | 72 (68·5%) |
| Chemotherapy alone | 23 (6·70%) | 16 (6·80%) | 7 (6·7%) |
| No chemotherapy or trastuzumab | 112 (32·7%) | 86 (36·2%) | 26 (24·8%) |
| Adjuvant ET | | | |
| Yes | 336 (98·2%) | 233 (98·4%) | 103 (98·1%) |
| Letrozole | 174 (50·9%) | 123 (51·9%) | 51 (48·6%) |
| Anastrozole | 139 (40·6%) | 91 (38·4%) | 48 (45·7%) |
| Exemestane | 1 (0·30%) | 1 (0·40%) | 0 (0·0%) |
| Tamoxifen | 7 (2·0%) | 7 (3·10%) | 0 (0·0%) |
| Sequential AI and tamoxifen | 15 (4·40%) | 11 (4·60%) | 4 (3·8%) |
| No adjuvant endocrine therapy | 4 (1·20%) | 2 (0·80%) | 2 (1·9%) |
| Unknown | 2 (0·60%) | 2 (0·80%) | 0 (0%) |

## Slide 4
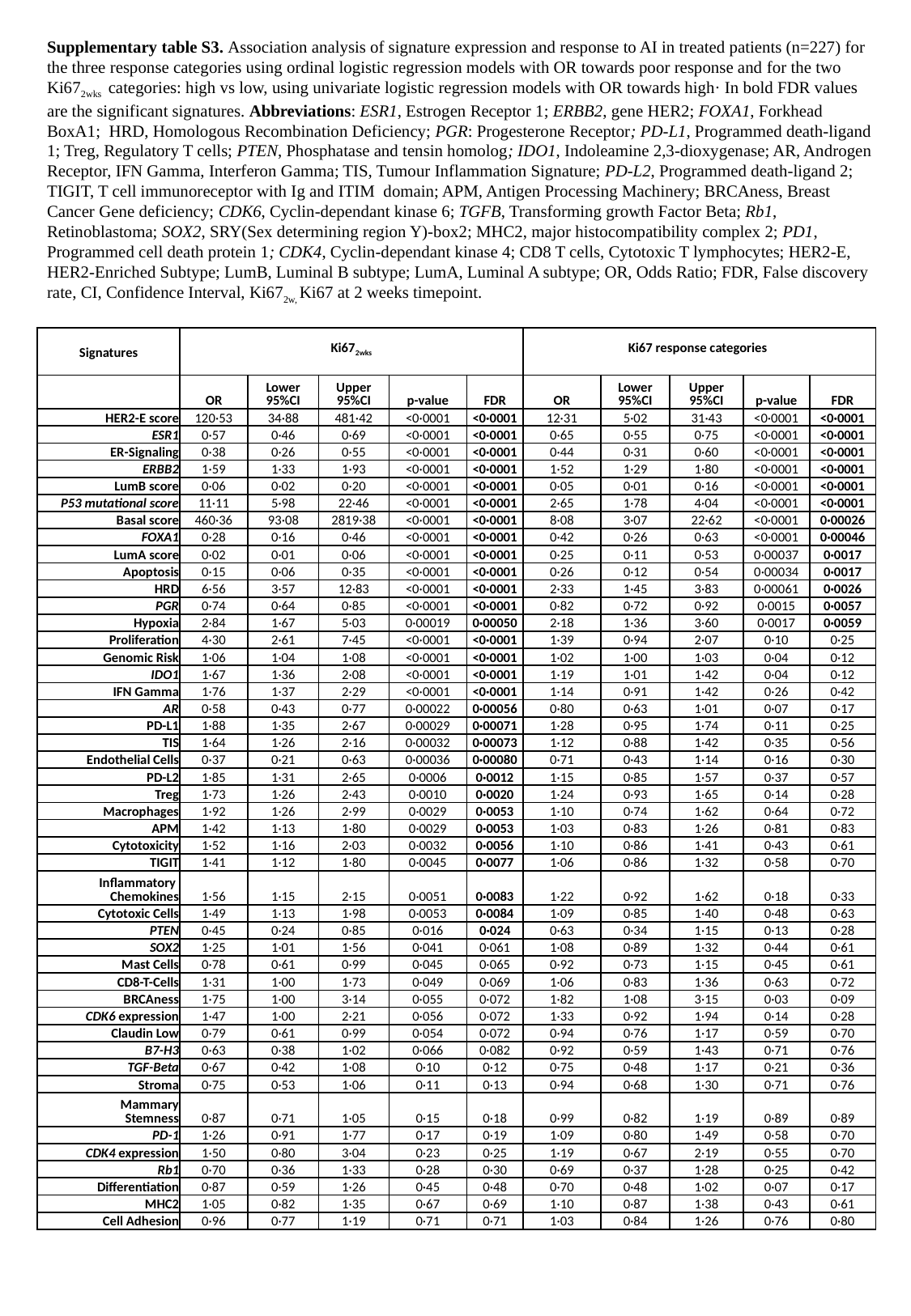

# Supplementary table S3. Association analysis of signature expression and response to AI in treated patients (n=227) for the three response categories using ordinal logistic regression models with OR towards poor response and for the two Ki672wks categories: high vs low, using univariate logistic regression models with OR towards high· In bold FDR values are the significant signatures. Abbreviations: ESR1, Estrogen Receptor 1; ERBB2, gene HER2; FOXA1, Forkhead BoxA1; HRD, Homologous Recombination Deficiency; PGR: Progesterone Receptor; PD-L1, Programmed death-ligand 1; Treg, Regulatory T cells; PTEN, Phosphatase and tensin homolog; IDO1, Indoleamine 2,3-dioxygenase; AR, Androgen Receptor, IFN Gamma, Interferon Gamma; TIS, Tumour Inflammation Signature; PD-L2, Programmed death-ligand 2; TIGIT, T cell immunoreceptor with Ig and ITIM domain; APM, Antigen Processing Machinery; BRCAness, Breast Cancer Gene deficiency; CDK6, Cyclin-dependant kinase 6; TGFB, Transforming growth Factor Beta; Rb1, Retinoblastoma; SOX2, SRY(Sex determining region Y)-box2; MHC2, major histocompatibility complex 2; PD1, Programmed cell death protein 1; CDK4, Cyclin-dependant kinase 4; CD8 T cells, Cytotoxic T lymphocytes; HER2-E, HER2-Enriched Subtype; LumB, Luminal B subtype; LumA, Luminal A subtype; OR, Odds Ratio; FDR, False discovery rate, CI, Confidence Interval, Ki672w, Ki67 at 2 weeks timepoint.
| Signatures | Ki672wks | | | | | Ki67 response categories | | | | |
| --- | --- | --- | --- | --- | --- | --- | --- | --- | --- | --- |
| | OR | Lower 95%CI | Upper 95%CI | p-value | FDR | OR | Lower 95%CI | Upper 95%CI | p-value | FDR |
| HER2-E score | 120·53 | 34·88 | 481·42 | <0·0001 | <0·0001 | 12·31 | 5·02 | 31·43 | <0·0001 | <0·0001 |
| ESR1 | 0·57 | 0·46 | 0·69 | <0·0001 | <0·0001 | 0·65 | 0·55 | 0·75 | <0·0001 | <0·0001 |
| ER-Signaling | 0·38 | 0·26 | 0·55 | <0·0001 | <0·0001 | 0·44 | 0·31 | 0·60 | <0·0001 | <0·0001 |
| ERBB2 | 1·59 | 1·33 | 1·93 | <0·0001 | <0·0001 | 1·52 | 1·29 | 1·80 | <0·0001 | <0·0001 |
| LumB score | 0·06 | 0·02 | 0·20 | <0·0001 | <0·0001 | 0·05 | 0·01 | 0·16 | <0·0001 | <0·0001 |
| P53 mutational score | 11·11 | 5·98 | 22·46 | <0·0001 | <0·0001 | 2·65 | 1·78 | 4·04 | <0·0001 | <0·0001 |
| Basal score | 460·36 | 93·08 | 2819·38 | <0·0001 | <0·0001 | 8·08 | 3·07 | 22·62 | <0·0001 | 0·00026 |
| FOXA1 | 0·28 | 0·16 | 0·46 | <0·0001 | <0·0001 | 0·42 | 0·26 | 0·63 | <0·0001 | 0·00046 |
| LumA score | 0·02 | 0·01 | 0·06 | <0·0001 | <0·0001 | 0·25 | 0·11 | 0·53 | 0·00037 | 0·0017 |
| Apoptosis | 0·15 | 0·06 | 0·35 | <0·0001 | <0·0001 | 0·26 | 0·12 | 0·54 | 0·00034 | 0·0017 |
| HRD | 6·56 | 3·57 | 12·83 | <0·0001 | <0·0001 | 2·33 | 1·45 | 3·83 | 0·00061 | 0·0026 |
| PGR | 0·74 | 0·64 | 0·85 | <0·0001 | <0·0001 | 0·82 | 0·72 | 0·92 | 0·0015 | 0·0057 |
| Hypoxia | 2·84 | 1·67 | 5·03 | 0·00019 | 0·00050 | 2·18 | 1·36 | 3·60 | 0·0017 | 0·0059 |
| Proliferation | 4·30 | 2·61 | 7·45 | <0·0001 | <0·0001 | 1·39 | 0·94 | 2·07 | 0·10 | 0·25 |
| Genomic Risk | 1·06 | 1·04 | 1·08 | <0·0001 | <0·0001 | 1·02 | 1·00 | 1·03 | 0·04 | 0·12 |
| IDO1 | 1·67 | 1·36 | 2·08 | <0·0001 | <0·0001 | 1·19 | 1·01 | 1·42 | 0·04 | 0·12 |
| IFN Gamma | 1·76 | 1·37 | 2·29 | <0·0001 | <0·0001 | 1·14 | 0·91 | 1·42 | 0·26 | 0·42 |
| AR | 0·58 | 0·43 | 0·77 | 0·00022 | 0·00056 | 0·80 | 0·63 | 1·01 | 0·07 | 0·17 |
| PD-L1 | 1·88 | 1·35 | 2·67 | 0·00029 | 0·00071 | 1·28 | 0·95 | 1·74 | 0·11 | 0·25 |
| TIS | 1·64 | 1·26 | 2·16 | 0·00032 | 0·00073 | 1·12 | 0·88 | 1·42 | 0·35 | 0·56 |
| Endothelial Cells | 0·37 | 0·21 | 0·63 | 0·00036 | 0·00080 | 0·71 | 0·43 | 1·14 | 0·16 | 0·30 |
| PD-L2 | 1·85 | 1·31 | 2·65 | 0·0006 | 0·0012 | 1·15 | 0·85 | 1·57 | 0·37 | 0·57 |
| Treg | 1·73 | 1·26 | 2·43 | 0·0010 | 0·0020 | 1·24 | 0·93 | 1·65 | 0·14 | 0·28 |
| Macrophages | 1·92 | 1·26 | 2·99 | 0·0029 | 0·0053 | 1·10 | 0·74 | 1·62 | 0·64 | 0·72 |
| APM | 1·42 | 1·13 | 1·80 | 0·0029 | 0·0053 | 1·03 | 0·83 | 1·26 | 0·81 | 0·83 |
| Cytotoxicity | 1·52 | 1·16 | 2·03 | 0·0032 | 0·0056 | 1·10 | 0·86 | 1·41 | 0·43 | 0·61 |
| TIGIT | 1·41 | 1·12 | 1·80 | 0·0045 | 0·0077 | 1·06 | 0·86 | 1·32 | 0·58 | 0·70 |
| Inflammatory Chemokines | 1·56 | 1·15 | 2·15 | 0·0051 | 0·0083 | 1·22 | 0·92 | 1·62 | 0·18 | 0·33 |
| Cytotoxic Cells | 1·49 | 1·13 | 1·98 | 0·0053 | 0·0084 | 1·09 | 0·85 | 1·40 | 0·48 | 0·63 |
| PTEN | 0·45 | 0·24 | 0·85 | 0·016 | 0·024 | 0·63 | 0·34 | 1·15 | 0·13 | 0·28 |
| SOX2 | 1·25 | 1·01 | 1·56 | 0·041 | 0·061 | 1·08 | 0·89 | 1·32 | 0·44 | 0·61 |
| Mast Cells | 0·78 | 0·61 | 0·99 | 0·045 | 0·065 | 0·92 | 0·73 | 1·15 | 0·45 | 0·61 |
| CD8-T-Cells | 1·31 | 1·00 | 1·73 | 0·049 | 0·069 | 1·06 | 0·83 | 1·36 | 0·63 | 0·72 |
| BRCAness | 1·75 | 1·00 | 3·14 | 0·055 | 0·072 | 1·82 | 1·08 | 3·15 | 0·03 | 0·09 |
| CDK6 expression | 1·47 | 1·00 | 2·21 | 0·056 | 0·072 | 1·33 | 0·92 | 1·94 | 0·14 | 0·28 |
| Claudin Low | 0·79 | 0·61 | 0·99 | 0·054 | 0·072 | 0·94 | 0·76 | 1·17 | 0·59 | 0·70 |
| B7-H3 | 0·63 | 0·38 | 1·02 | 0·066 | 0·082 | 0·92 | 0·59 | 1·43 | 0·71 | 0·76 |
| TGF-Beta | 0·67 | 0·42 | 1·08 | 0·10 | 0·12 | 0·75 | 0·48 | 1·17 | 0·21 | 0·36 |
| Stroma | 0·75 | 0·53 | 1·06 | 0·11 | 0·13 | 0·94 | 0·68 | 1·30 | 0·71 | 0·76 |
| Mammary Stemness | 0·87 | 0·71 | 1·05 | 0·15 | 0·18 | 0·99 | 0·82 | 1·19 | 0·89 | 0·89 |
| PD-1 | 1·26 | 0·91 | 1·77 | 0·17 | 0·19 | 1·09 | 0·80 | 1·49 | 0·58 | 0·70 |
| CDK4 expression | 1·50 | 0·80 | 3·04 | 0·23 | 0·25 | 1·19 | 0·67 | 2·19 | 0·55 | 0·70 |
| Rb1 | 0·70 | 0·36 | 1·33 | 0·28 | 0·30 | 0·69 | 0·37 | 1·28 | 0·25 | 0·42 |
| Differentiation | 0·87 | 0·59 | 1·26 | 0·45 | 0·48 | 0·70 | 0·48 | 1·02 | 0·07 | 0·17 |
| MHC2 | 1·05 | 0·82 | 1·35 | 0·67 | 0·69 | 1·10 | 0·87 | 1·38 | 0·43 | 0·61 |
| Cell Adhesion | 0·96 | 0·77 | 1·19 | 0·71 | 0·71 | 1·03 | 0·84 | 1·26 | 0·76 | 0·80 |

## Slide 5
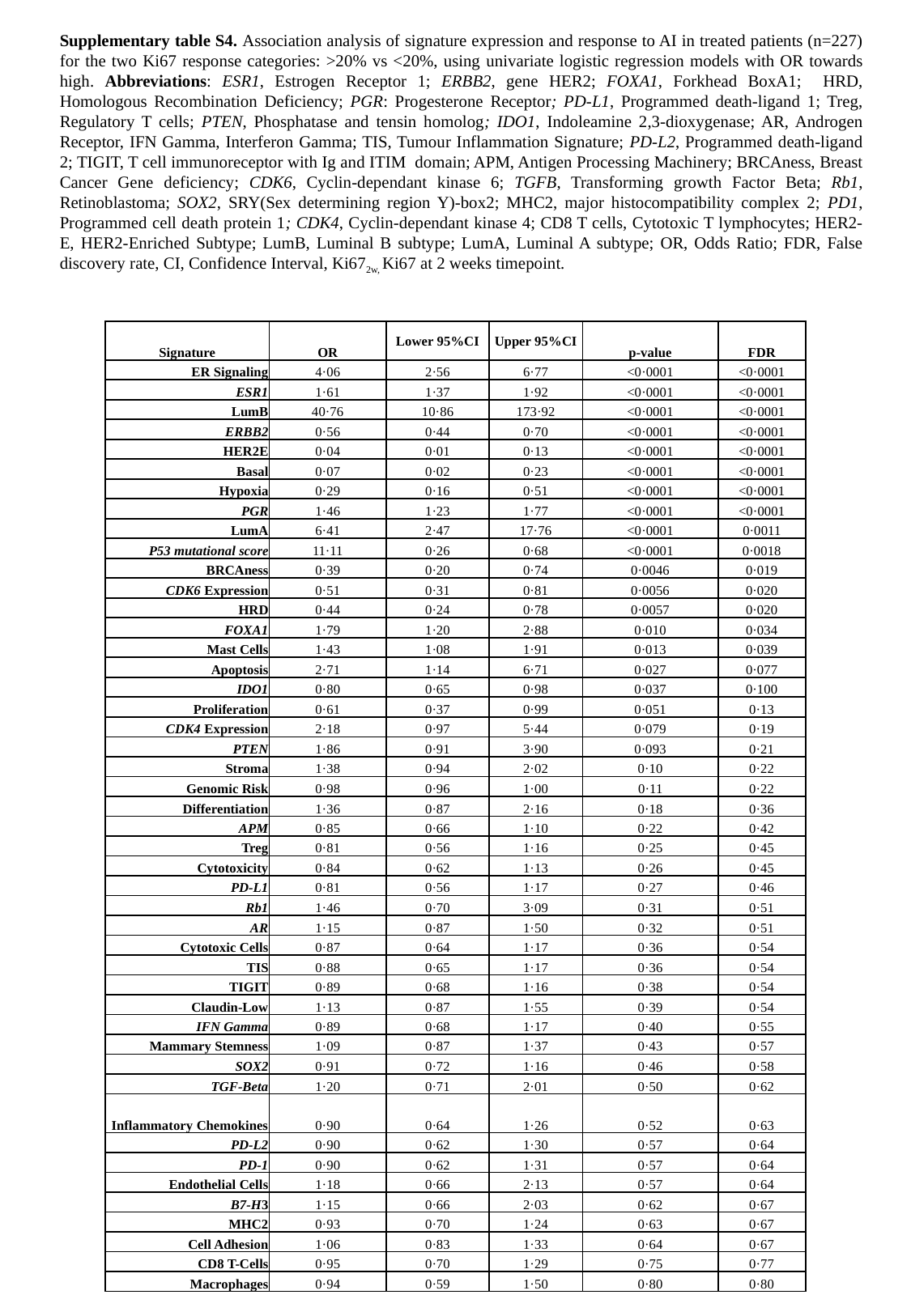

Supplementary table S4. Association analysis of signature expression and response to AI in treated patients (n=227) for the two Ki67 response categories: >20% vs <20%, using univariate logistic regression models with OR towards high. Abbreviations: ESR1, Estrogen Receptor 1; ERBB2, gene HER2; FOXA1, Forkhead BoxA1; HRD, Homologous Recombination Deficiency; PGR: Progesterone Receptor; PD-L1, Programmed death-ligand 1; Treg, Regulatory T cells; PTEN, Phosphatase and tensin homolog; IDO1, Indoleamine 2,3-dioxygenase; AR, Androgen Receptor, IFN Gamma, Interferon Gamma; TIS, Tumour Inflammation Signature; PD-L2, Programmed death-ligand 2; TIGIT, T cell immunoreceptor with Ig and ITIM domain; APM, Antigen Processing Machinery; BRCAness, Breast Cancer Gene deficiency; CDK6, Cyclin-dependant kinase 6; TGFB, Transforming growth Factor Beta; Rb1, Retinoblastoma; SOX2, SRY(Sex determining region Y)-box2; MHC2, major histocompatibility complex 2; PD1, Programmed cell death protein 1; CDK4, Cyclin-dependant kinase 4; CD8 T cells, Cytotoxic T lymphocytes; HER2-E, HER2-Enriched Subtype; LumB, Luminal B subtype; LumA, Luminal A subtype; OR, Odds Ratio; FDR, False discovery rate, CI, Confidence Interval, Ki672w, Ki67 at 2 weeks timepoint.
| Signature | OR | Lower 95%CI | Upper 95%CI | p-value | FDR |
| --- | --- | --- | --- | --- | --- |
| ER Signaling | 4·06 | 2·56 | 6·77 | <0·0001 | <0·0001 |
| ESR1 | 1·61 | 1·37 | 1·92 | <0·0001 | <0·0001 |
| LumB | 40·76 | 10·86 | 173·92 | <0·0001 | <0·0001 |
| ERBB2 | 0·56 | 0·44 | 0·70 | <0·0001 | <0·0001 |
| HER2E | 0·04 | 0·01 | 0·13 | <0·0001 | <0·0001 |
| Basal | 0·07 | 0·02 | 0·23 | <0·0001 | <0·0001 |
| Hypoxia | 0·29 | 0·16 | 0·51 | <0·0001 | <0·0001 |
| PGR | 1·46 | 1·23 | 1·77 | <0·0001 | <0·0001 |
| LumA | 6·41 | 2·47 | 17·76 | <0·0001 | 0·0011 |
| P53 mutational score | 11·11 | 0·26 | 0·68 | <0·0001 | 0·0018 |
| BRCAness | 0·39 | 0·20 | 0·74 | 0·0046 | 0·019 |
| CDK6 Expression | 0·51 | 0·31 | 0·81 | 0·0056 | 0·020 |
| HRD | 0·44 | 0·24 | 0·78 | 0·0057 | 0·020 |
| FOXA1 | 1·79 | 1·20 | 2·88 | 0·010 | 0·034 |
| Mast Cells | 1·43 | 1·08 | 1·91 | 0·013 | 0·039 |
| Apoptosis | 2·71 | 1·14 | 6·71 | 0·027 | 0·077 |
| IDO1 | 0·80 | 0·65 | 0·98 | 0·037 | 0·100 |
| Proliferation | 0·61 | 0·37 | 0·99 | 0·051 | 0·13 |
| CDK4 Expression | 2·18 | 0·97 | 5·44 | 0·079 | 0·19 |
| PTEN | 1·86 | 0·91 | 3·90 | 0·093 | 0·21 |
| Stroma | 1·38 | 0·94 | 2·02 | 0·10 | 0·22 |
| Genomic Risk | 0·98 | 0·96 | 1·00 | 0·11 | 0·22 |
| Differentiation | 1·36 | 0·87 | 2·16 | 0·18 | 0·36 |
| APM | 0·85 | 0·66 | 1·10 | 0·22 | 0·42 |
| Treg | 0·81 | 0·56 | 1·16 | 0·25 | 0·45 |
| Cytotoxicity | 0·84 | 0·62 | 1·13 | 0·26 | 0·45 |
| PD-L1 | 0·81 | 0·56 | 1·17 | 0·27 | 0·46 |
| Rb1 | 1·46 | 0·70 | 3·09 | 0·31 | 0·51 |
| AR | 1·15 | 0·87 | 1·50 | 0·32 | 0·51 |
| Cytotoxic Cells | 0·87 | 0·64 | 1·17 | 0·36 | 0·54 |
| TIS | 0·88 | 0·65 | 1·17 | 0·36 | 0·54 |
| TIGIT | 0·89 | 0·68 | 1·16 | 0·38 | 0·54 |
| Claudin-Low | 1·13 | 0·87 | 1·55 | 0·39 | 0·54 |
| IFN Gamma | 0·89 | 0·68 | 1·17 | 0·40 | 0·55 |
| Mammary Stemness | 1·09 | 0·87 | 1·37 | 0·43 | 0·57 |
| SOX2 | 0·91 | 0·72 | 1·16 | 0·46 | 0·58 |
| TGF-Beta | 1·20 | 0·71 | 2·01 | 0·50 | 0·62 |
| Inflammatory Chemokines | 0·90 | 0·64 | 1·26 | 0·52 | 0·63 |
| PD-L2 | 0·90 | 0·62 | 1·30 | 0·57 | 0·64 |
| PD-1 | 0·90 | 0·62 | 1·31 | 0·57 | 0·64 |
| Endothelial Cells | 1·18 | 0·66 | 2·13 | 0·57 | 0·64 |
| B7-H3 | 1·15 | 0·66 | 2·03 | 0·62 | 0·67 |
| MHC2 | 0·93 | 0·70 | 1·24 | 0·63 | 0·67 |
| Cell Adhesion | 1·06 | 0·83 | 1·33 | 0·64 | 0·67 |
| CD8 T-Cells | 0·95 | 0·70 | 1·29 | 0·75 | 0·77 |
| Macrophages | 0·94 | 0·59 | 1·50 | 0·80 | 0·80 |

## Slide 6
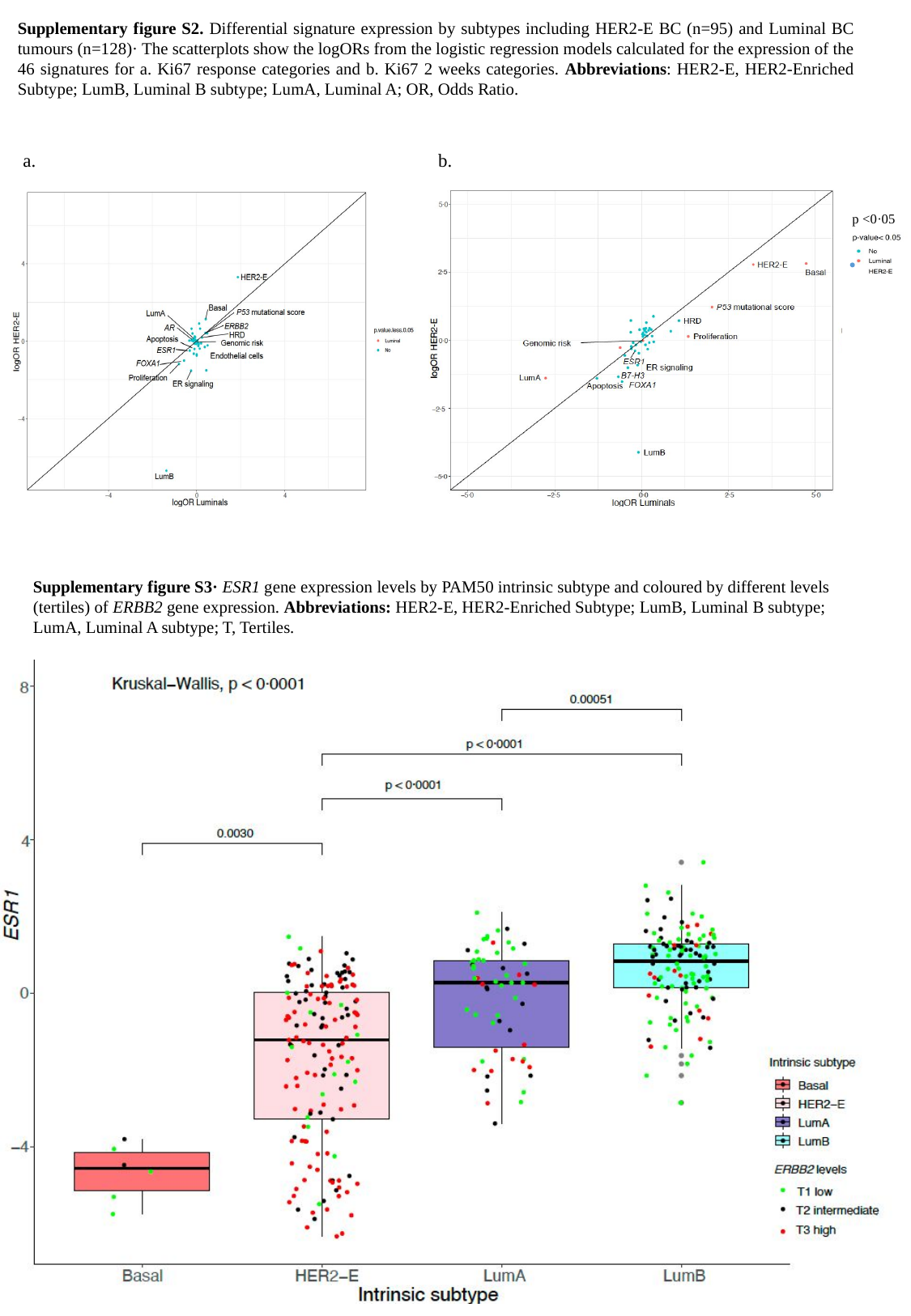

Supplementary figure S2. Differential signature expression by subtypes including HER2-E BC (n=95) and Luminal BC tumours (n=128)· The scatterplots show the logORs from the logistic regression models calculated for the expression of the 46 signatures for a. Ki67 response categories and b. Ki67 2 weeks categories. Abbreviations: HER2-E, HER2-Enriched Subtype; LumB, Luminal B subtype; LumA, Luminal A; OR, Odds Ratio.
a.
b.
p <0·05
Supplementary figure S3· ESR1 gene expression levels by PAM50 intrinsic subtype and coloured by different levels (tertiles) of ERBB2 gene expression. Abbreviations: HER2-E, HER2-Enriched Subtype; LumB, Luminal B subtype; LumA, Luminal A subtype; T, Tertiles.

## Slide 7
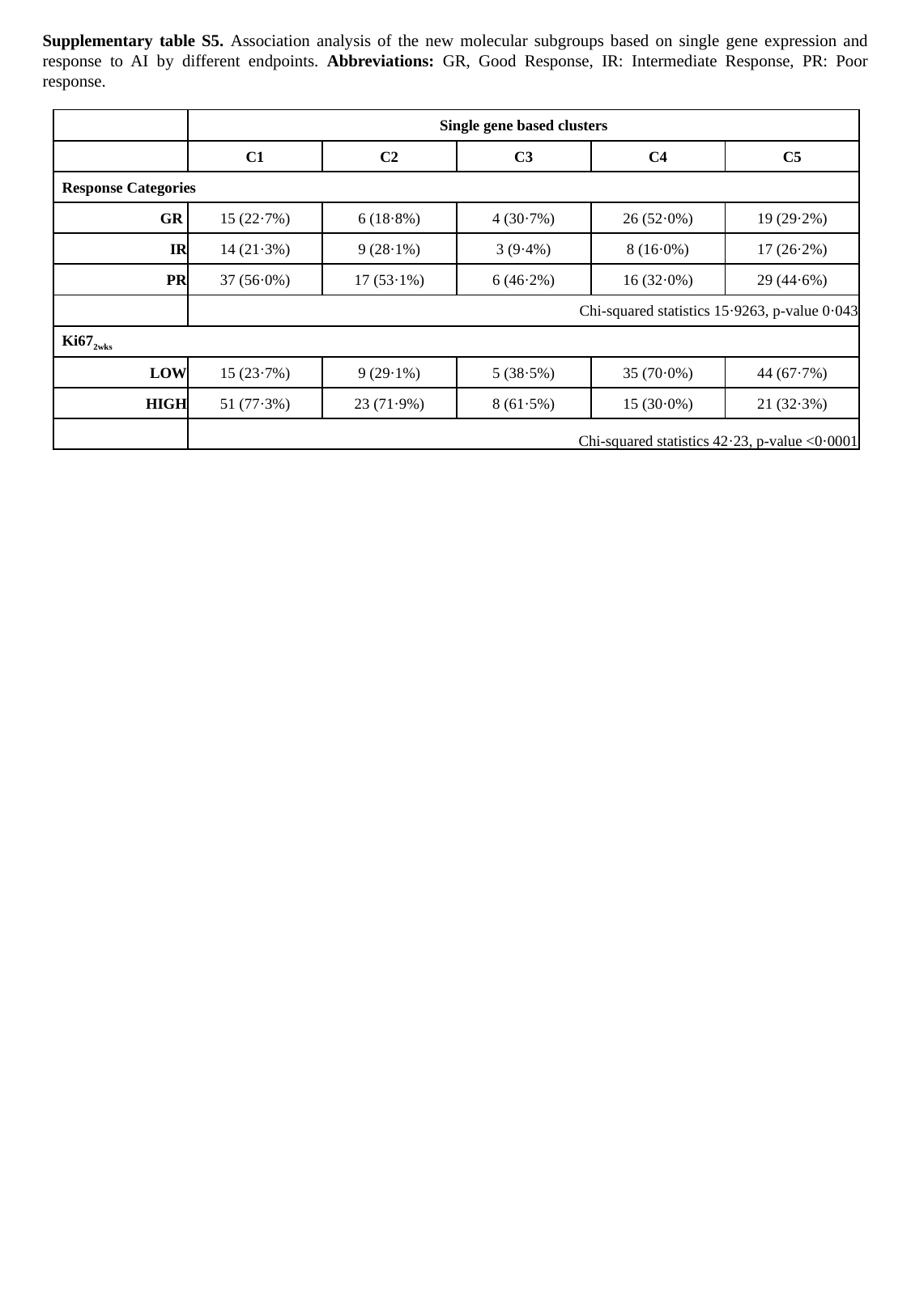

Supplementary table S5. Association analysis of the new molecular subgroups based on single gene expression and response to AI by different endpoints. Abbreviations: GR, Good Response, IR: Intermediate Response, PR: Poor response.
| | Single gene based clusters | | | | |
| --- | --- | --- | --- | --- | --- |
| | C1 | C2 | C3 | C4 | C5 |
| Response Categories | | | | | |
| GR | 15 (22·7%) | 6 (18·8%) | 4 (30·7%) | 26 (52·0%) | 19 (29·2%) |
| IR | 14 (21·3%) | 9 (28·1%) | 3 (9·4%) | 8 (16·0%) | 17 (26·2%) |
| PR | 37 (56·0%) | 17 (53·1%) | 6 (46·2%) | 16 (32·0%) | 29 (44·6%) |
| | Chi-squared statistics 15·9263, p-value 0·043 | | | | |
| Ki672wks | | | | | |
| LOW | 15 (23·7%) | 9 (29·1%) | 5 (38·5%) | 35 (70·0%) | 44 (67·7%) |
| HIGH | 51 (77·3%) | 23 (71·9%) | 8 (61·5%) | 15 (30·0%) | 21 (32·3%) |
| | Chi-squared statistics 42·23, p-value <0·0001 | | | | |

## Slide 8
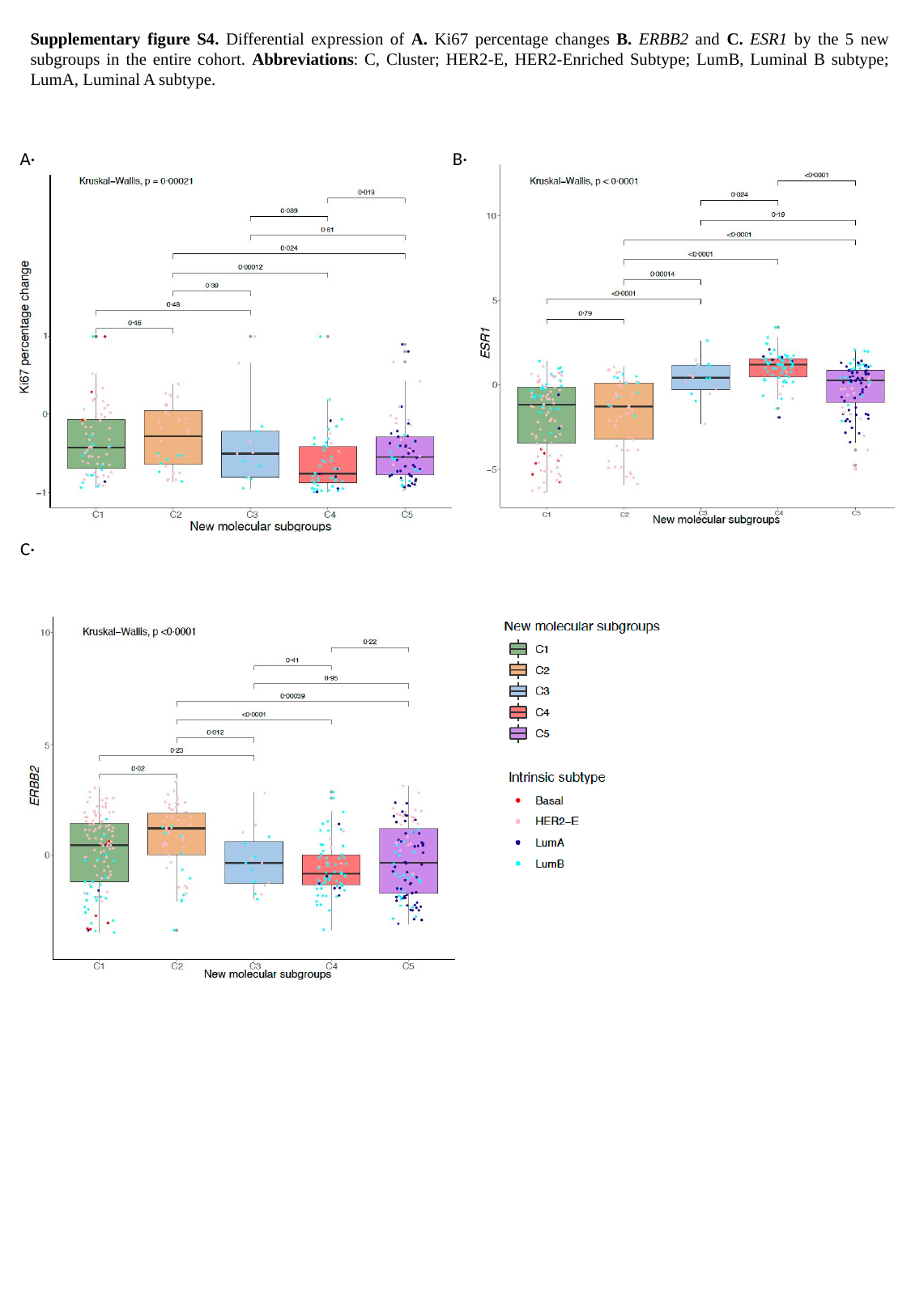

Supplementary figure S4. Differential expression of A. Ki67 percentage changes B. ERBB2 and C. ESR1 by the 5 new subgroups in the entire cohort. Abbreviations: C, Cluster; HER2-E, HER2-Enriched Subtype; LumB, Luminal B subtype; LumA, Luminal A subtype.
 A·
 B·
 C·

## Slide 9
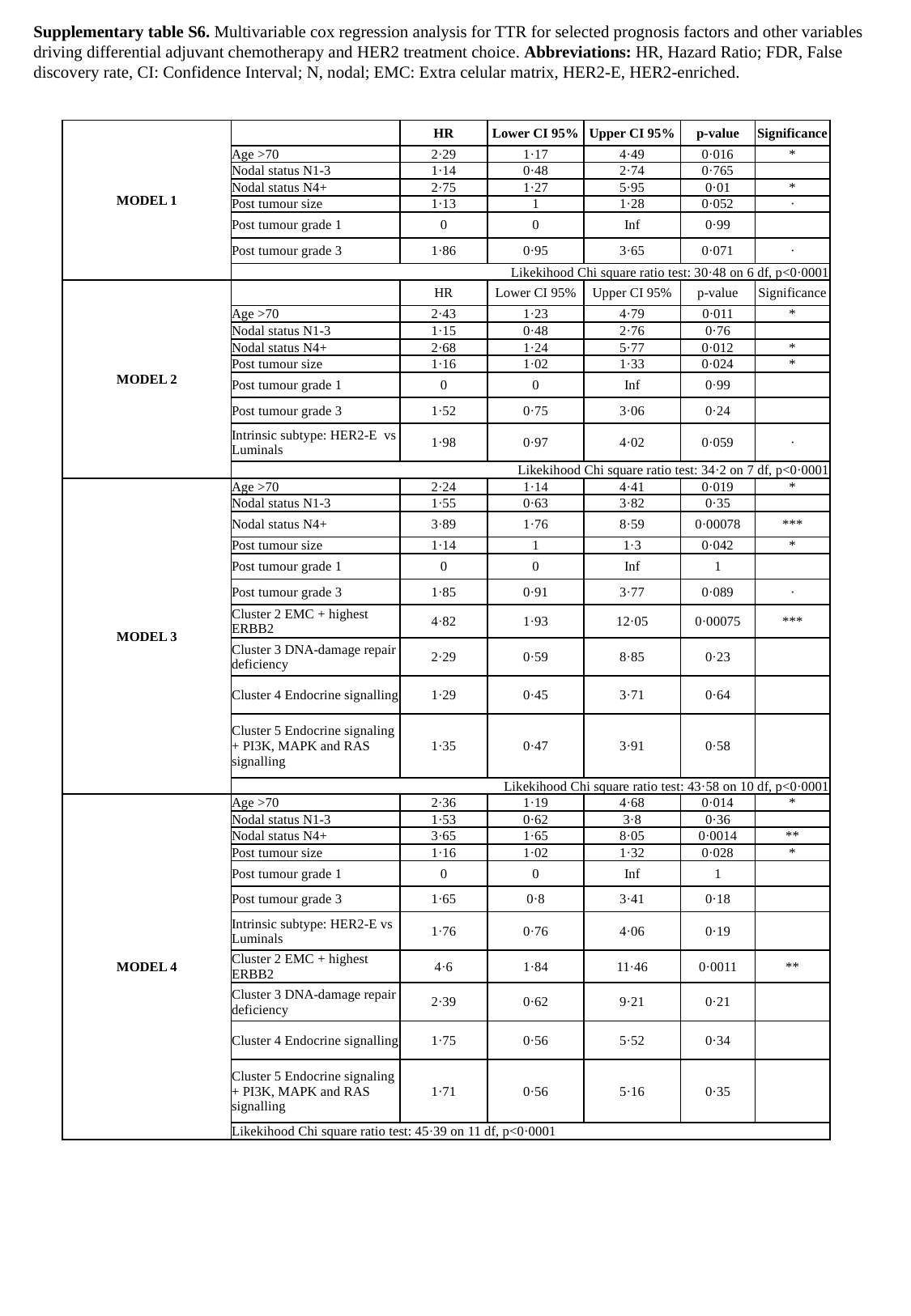

Supplementary table S6. Multivariable cox regression analysis for TTR for selected prognosis factors and other variables driving differential adjuvant chemotherapy and HER2 treatment choice. Abbreviations: HR, Hazard Ratio; FDR, False discovery rate, CI: Confidence Interval; N, nodal; EMC: Extra celular matrix, HER2-E, HER2-enriched.
| MODEL 1 | | HR | Lower CI 95% | Upper CI 95% | p-value | Significance |
| --- | --- | --- | --- | --- | --- | --- |
| | Age >70 | 2·29 | 1·17 | 4·49 | 0·016 | \* |
| | Nodal status N1-3 | 1·14 | 0·48 | 2·74 | 0·765 | |
| | Nodal status N4+ | 2·75 | 1·27 | 5·95 | 0·01 | \* |
| | Post tumour size | 1·13 | 1 | 1·28 | 0·052 | · |
| | Post tumour grade 1 | 0 | 0 | Inf | 0·99 | |
| | Post tumour grade 3 | 1·86 | 0·95 | 3·65 | 0·071 | · |
| | Likekihood Chi square ratio test: 30·48 on 6 df, p<0·0001 | | | | | |
| MODEL 2 | | HR | Lower CI 95% | Upper CI 95% | p-value | Significance |
| | Age >70 | 2·43 | 1·23 | 4·79 | 0·011 | \* |
| | Nodal status N1-3 | 1·15 | 0·48 | 2·76 | 0·76 | |
| | Nodal status N4+ | 2·68 | 1·24 | 5·77 | 0·012 | \* |
| | Post tumour size | 1·16 | 1·02 | 1·33 | 0·024 | \* |
| | Post tumour grade 1 | 0 | 0 | Inf | 0·99 | |
| | Post tumour grade 3 | 1·52 | 0·75 | 3·06 | 0·24 | |
| | Intrinsic subtype: HER2-E vs Luminals | 1·98 | 0·97 | 4·02 | 0·059 | · |
| | Likekihood Chi square ratio test: 34·2 on 7 df, p<0·0001 | | | | | |
| MODEL 3 | Age >70 | 2·24 | 1·14 | 4·41 | 0·019 | \* |
| | Nodal status N1-3 | 1·55 | 0·63 | 3·82 | 0·35 | |
| | Nodal status N4+ | 3·89 | 1·76 | 8·59 | 0·00078 | \*\*\* |
| | Post tumour size | 1·14 | 1 | 1·3 | 0·042 | \* |
| | Post tumour grade 1 | 0 | 0 | Inf | 1 | |
| | Post tumour grade 3 | 1·85 | 0·91 | 3·77 | 0·089 | · |
| | Cluster 2 EMC + highest ERBB2 | 4·82 | 1·93 | 12·05 | 0·00075 | \*\*\* |
| | Cluster 3 DNA-damage repair deficiency | 2·29 | 0·59 | 8·85 | 0·23 | |
| | Cluster 4 Endocrine signalling | 1·29 | 0·45 | 3·71 | 0·64 | |
| | Cluster 5 Endocrine signaling + PI3K, MAPK and RAS signalling | 1·35 | 0·47 | 3·91 | 0·58 | |
| | Likekihood Chi square ratio test: 43·58 on 10 df, p<0·0001 | | | | | |
| MODEL 4 | Age >70 | 2·36 | 1·19 | 4·68 | 0·014 | \* |
| | Nodal status N1-3 | 1·53 | 0·62 | 3·8 | 0·36 | |
| | Nodal status N4+ | 3·65 | 1·65 | 8·05 | 0·0014 | \*\* |
| | Post tumour size | 1·16 | 1·02 | 1·32 | 0·028 | \* |
| | Post tumour grade 1 | 0 | 0 | Inf | 1 | |
| | Post tumour grade 3 | 1·65 | 0·8 | 3·41 | 0·18 | |
| | Intrinsic subtype: HER2-E vs Luminals | 1·76 | 0·76 | 4·06 | 0·19 | |
| | Cluster 2 EMC + highest ERBB2 | 4·6 | 1·84 | 11·46 | 0·0011 | \*\* |
| | Cluster 3 DNA-damage repair deficiency | 2·39 | 0·62 | 9·21 | 0·21 | |
| | Cluster 4 Endocrine signalling | 1·75 | 0·56 | 5·52 | 0·34 | |
| | Cluster 5 Endocrine signaling + PI3K, MAPK and RAS signalling | 1·71 | 0·56 | 5·16 | 0·35 | |
| | Likekihood Chi square ratio test: 45·39 on 11 df, p<0·0001 | | | | | |

## Slide 10
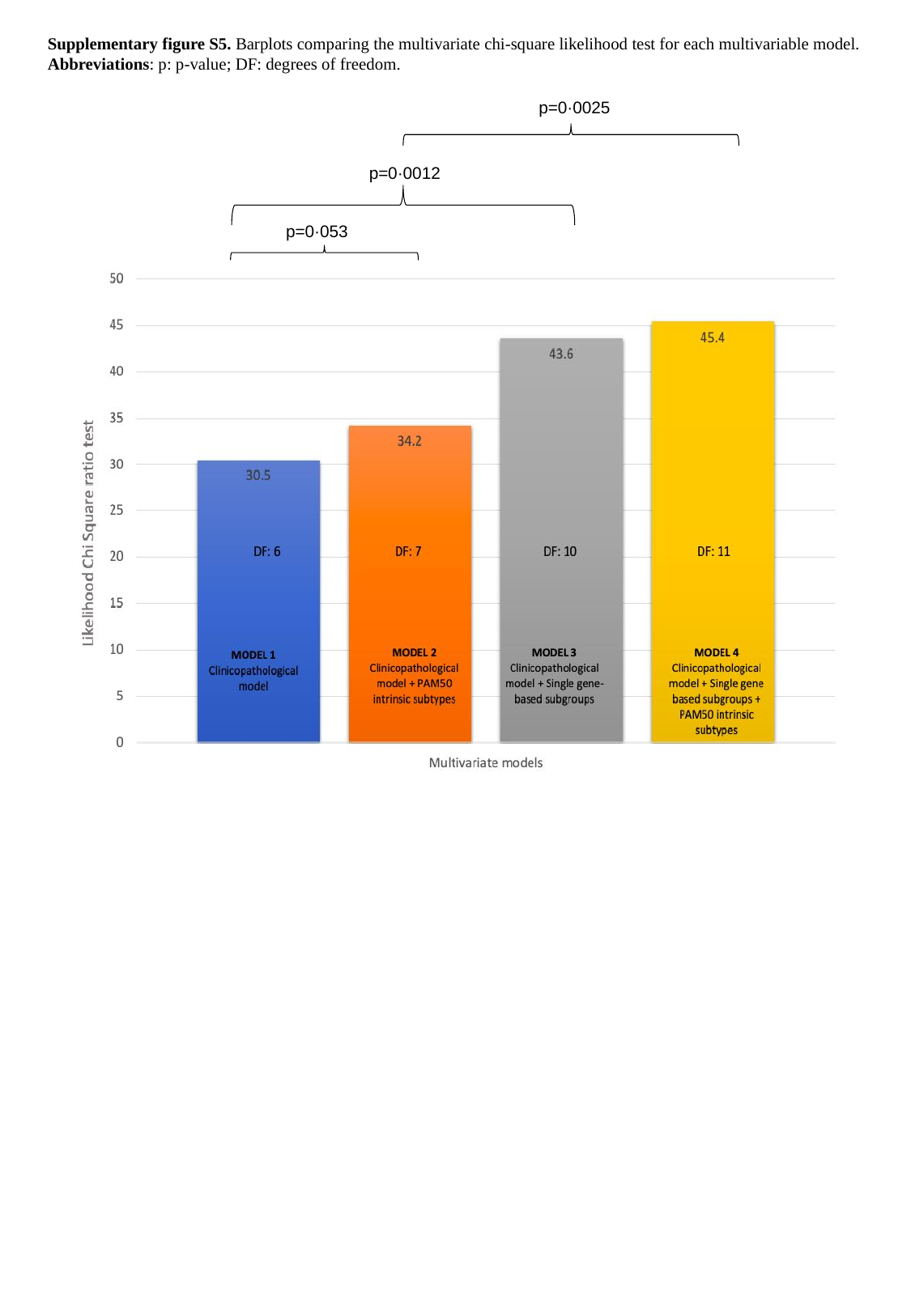

Supplementary figure S5. Barplots comparing the multivariate chi-square likelihood test for each multivariable model. Abbreviations: p: p-value; DF: degrees of freedom.
p=0·0025
p=0·0012
p=0·053

## Slide 11
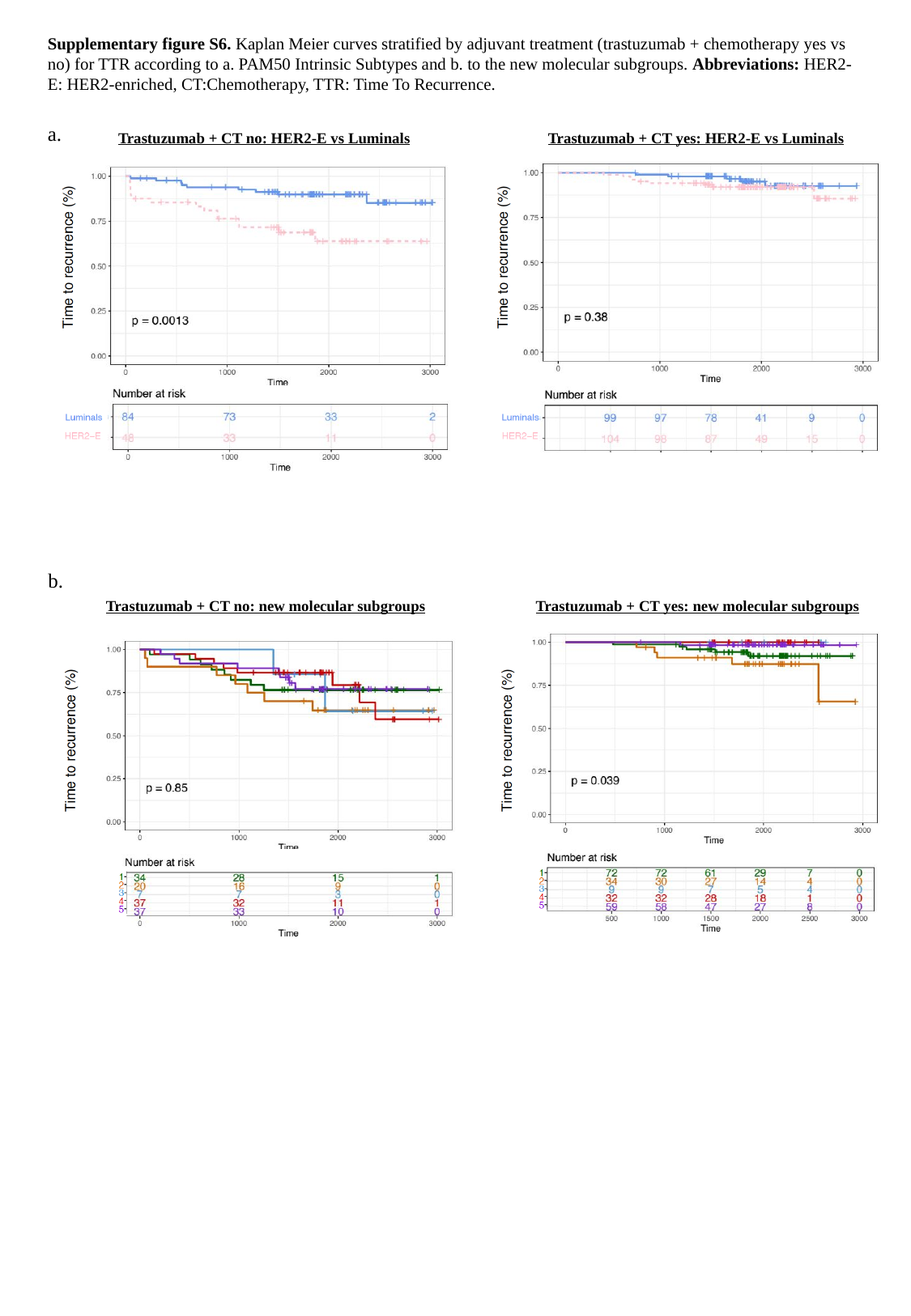

Supplementary figure S6. Kaplan Meier curves stratified by adjuvant treatment (trastuzumab + chemotherapy yes vs no) for TTR according to a. PAM50 Intrinsic Subtypes and b. to the new molecular subgroups. Abbreviations: HER2-E: HER2-enriched, CT:Chemotherapy, TTR: Time To Recurrence.
Trastuzumab + CT no: HER2-E vs Luminals	 Trastuzumab + CT yes: HER2-E vs Luminals
a.
b.
Trastuzumab + CT no: new molecular subgroups	 Trastuzumab + CT yes: new molecular subgroups

## Slide 12
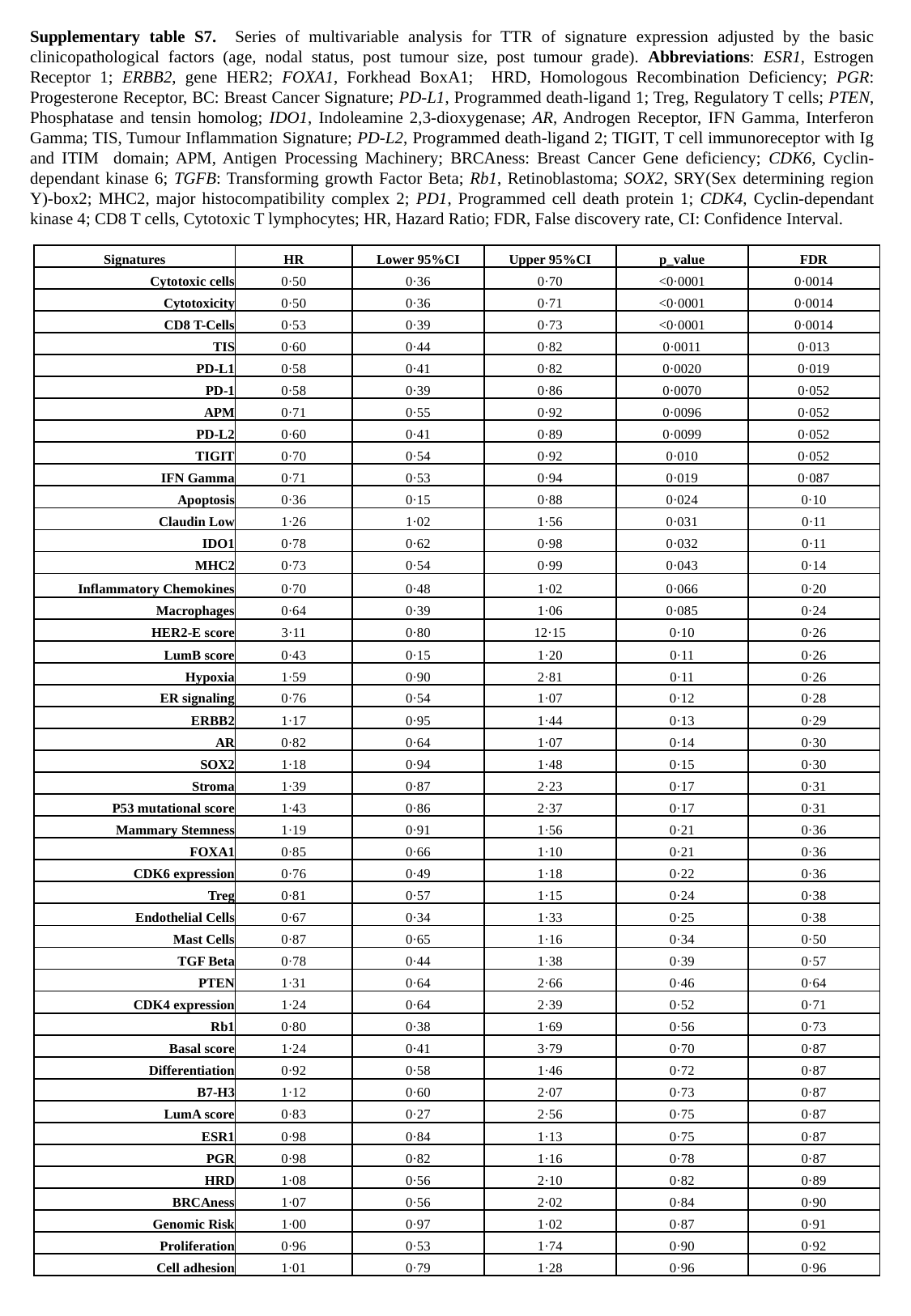

Supplementary table S7. Series of multivariable analysis for TTR of signature expression adjusted by the basic clinicopathological factors (age, nodal status, post tumour size, post tumour grade). Abbreviations: ESR1, Estrogen Receptor 1; ERBB2, gene HER2; FOXA1, Forkhead BoxA1; HRD, Homologous Recombination Deficiency; PGR: Progesterone Receptor, BC: Breast Cancer Signature; PD-L1, Programmed death-ligand 1; Treg, Regulatory T cells; PTEN, Phosphatase and tensin homolog; IDO1, Indoleamine 2,3-dioxygenase; AR, Androgen Receptor, IFN Gamma, Interferon Gamma; TIS, Tumour Inflammation Signature; PD-L2, Programmed death-ligand 2; TIGIT, T cell immunoreceptor with Ig and ITIM domain; APM, Antigen Processing Machinery; BRCAness: Breast Cancer Gene deficiency; CDK6, Cyclin-dependant kinase 6; TGFB: Transforming growth Factor Beta; Rb1, Retinoblastoma; SOX2, SRY(Sex determining region Y)-box2; MHC2, major histocompatibility complex 2; PD1, Programmed cell death protein 1; CDK4, Cyclin-dependant kinase 4; CD8 T cells, Cytotoxic T lymphocytes; HR, Hazard Ratio; FDR, False discovery rate, CI: Confidence Interval.
| Signatures | HR | Lower 95%CI | Upper 95%CI | p\_value | FDR |
| --- | --- | --- | --- | --- | --- |
| Cytotoxic cells | 0·50 | 0·36 | 0·70 | <0·0001 | 0·0014 |
| Cytotoxicity | 0·50 | 0·36 | 0·71 | <0·0001 | 0·0014 |
| CD8 T-Cells | 0·53 | 0·39 | 0·73 | <0·0001 | 0·0014 |
| TIS | 0·60 | 0·44 | 0·82 | 0·0011 | 0·013 |
| PD-L1 | 0·58 | 0·41 | 0·82 | 0·0020 | 0·019 |
| PD-1 | 0·58 | 0·39 | 0·86 | 0·0070 | 0·052 |
| APM | 0·71 | 0·55 | 0·92 | 0·0096 | 0·052 |
| PD-L2 | 0·60 | 0·41 | 0·89 | 0·0099 | 0·052 |
| TIGIT | 0·70 | 0·54 | 0·92 | 0·010 | 0·052 |
| IFN Gamma | 0·71 | 0·53 | 0·94 | 0·019 | 0·087 |
| Apoptosis | 0·36 | 0·15 | 0·88 | 0·024 | 0·10 |
| Claudin Low | 1·26 | 1·02 | 1·56 | 0·031 | 0·11 |
| IDO1 | 0·78 | 0·62 | 0·98 | 0·032 | 0·11 |
| MHC2 | 0·73 | 0·54 | 0·99 | 0·043 | 0·14 |
| Inflammatory Chemokines | 0·70 | 0·48 | 1·02 | 0·066 | 0·20 |
| Macrophages | 0·64 | 0·39 | 1·06 | 0·085 | 0·24 |
| HER2-E score | 3·11 | 0·80 | 12·15 | 0·10 | 0·26 |
| LumB score | 0·43 | 0·15 | 1·20 | 0·11 | 0·26 |
| Hypoxia | 1·59 | 0·90 | 2·81 | 0·11 | 0·26 |
| ER signaling | 0·76 | 0·54 | 1·07 | 0·12 | 0·28 |
| ERBB2 | 1·17 | 0·95 | 1·44 | 0·13 | 0·29 |
| AR | 0·82 | 0·64 | 1·07 | 0·14 | 0·30 |
| SOX2 | 1·18 | 0·94 | 1·48 | 0·15 | 0·30 |
| Stroma | 1·39 | 0·87 | 2·23 | 0·17 | 0·31 |
| P53 mutational score | 1·43 | 0·86 | 2·37 | 0·17 | 0·31 |
| Mammary Stemness | 1·19 | 0·91 | 1·56 | 0·21 | 0·36 |
| FOXA1 | 0·85 | 0·66 | 1·10 | 0·21 | 0·36 |
| CDK6 expression | 0·76 | 0·49 | 1·18 | 0·22 | 0·36 |
| Treg | 0·81 | 0·57 | 1·15 | 0·24 | 0·38 |
| Endothelial Cells | 0·67 | 0·34 | 1·33 | 0·25 | 0·38 |
| Mast Cells | 0·87 | 0·65 | 1·16 | 0·34 | 0·50 |
| TGF Beta | 0·78 | 0·44 | 1·38 | 0·39 | 0·57 |
| PTEN | 1·31 | 0·64 | 2·66 | 0·46 | 0·64 |
| CDK4 expression | 1·24 | 0·64 | 2·39 | 0·52 | 0·71 |
| Rb1 | 0·80 | 0·38 | 1·69 | 0·56 | 0·73 |
| Basal score | 1·24 | 0·41 | 3·79 | 0·70 | 0·87 |
| Differentiation | 0·92 | 0·58 | 1·46 | 0·72 | 0·87 |
| B7-H3 | 1·12 | 0·60 | 2·07 | 0·73 | 0·87 |
| LumA score | 0·83 | 0·27 | 2·56 | 0·75 | 0·87 |
| ESR1 | 0·98 | 0·84 | 1·13 | 0·75 | 0·87 |
| PGR | 0·98 | 0·82 | 1·16 | 0·78 | 0·87 |
| HRD | 1·08 | 0·56 | 2·10 | 0·82 | 0·89 |
| BRCAness | 1·07 | 0·56 | 2·02 | 0·84 | 0·90 |
| Genomic Risk | 1·00 | 0·97 | 1·02 | 0·87 | 0·91 |
| Proliferation | 0·96 | 0·53 | 1·74 | 0·90 | 0·92 |
| Cell adhesion | 1·01 | 0·79 | 1·28 | 0·96 | 0·96 |

## Slide 13
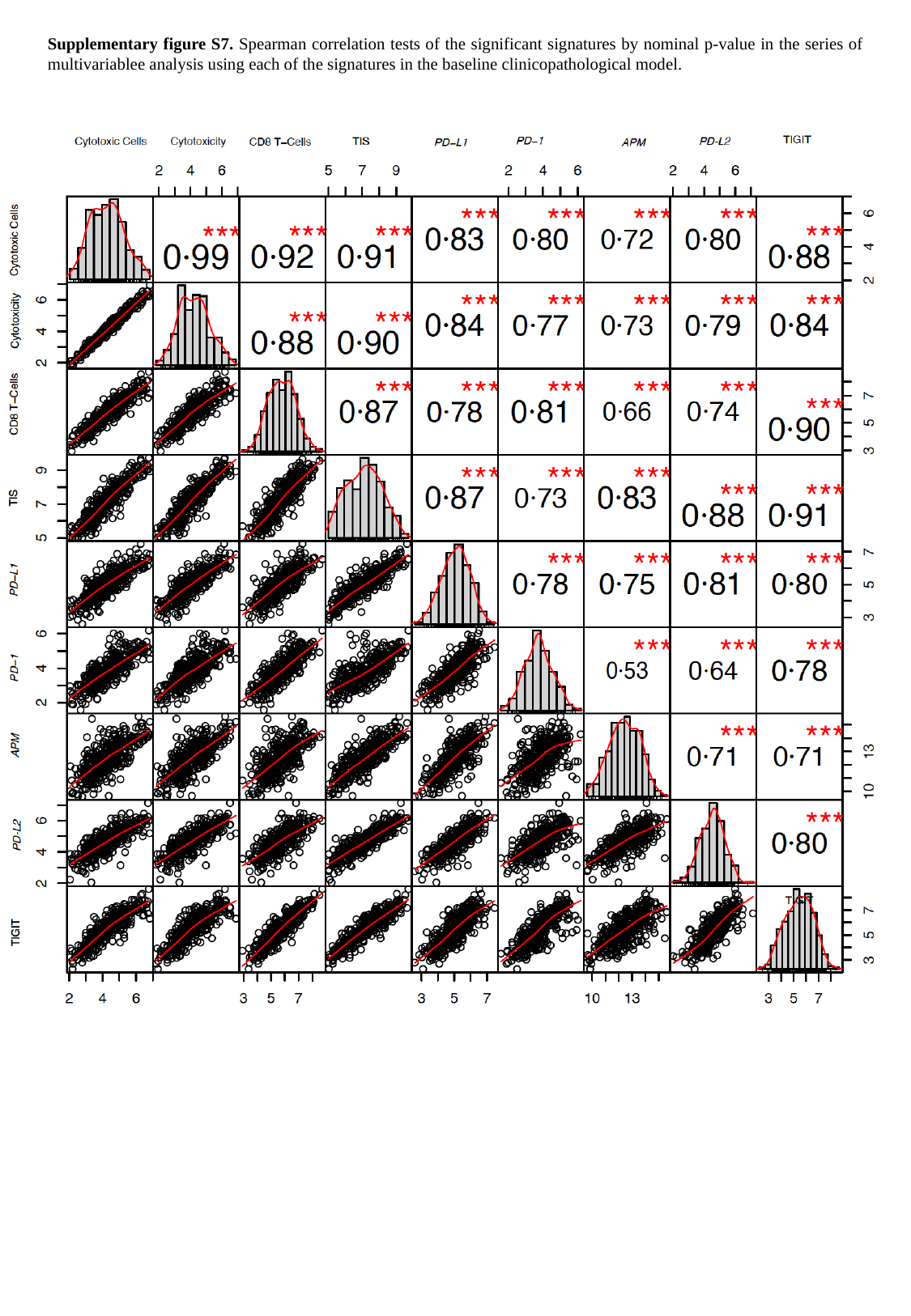

Supplementary figure S7. Spearman correlation tests of the significant signatures by nominal p-value in the series of multivariablee analysis using each of the signatures in the baseline clinicopathological model.
