# Supplementary material for: HER2-enriched subtype and novel molecular subgroups drive aromatase inhibitor resistance and an increased risk of relapse in early ER+/HER2+ breast cancer
Source: eBioMedicine. 2022 Aug 16;83:104205. doi: 10.1016/j.ebiom.2022.104205 (PMC9482930; doi:10.1016/j.ebiom.2022.104205)
Supplement: Supplementary file 3 [file mmc3.docx]

**Caption for supplementary material**

**Supplementary table POETIC CONSORTIA**. Group Author list of POETIC CONSORTIA.

**Supplementary figure S1.** Consort diagram of the study. **Abbreviations**: ER+, estrogen receptor positive; BC, Breast Cancer; IIT, insufficient infiltrating tumour.

**Supplementary table S1.** Breast Cancer 360 Biological signatures.

**Supplementary table S2.** Demography of the study population. **Abbreviations**: n: number; G: Grade; ILC: Invasive lobular carcinoma; IDC: Invasive ductal carcinoma; ER, Estrogen receptor; PgR, Progesterone receptor, ET, Endocrine Therapy.

**Supplementary table S3.** Association analysis of signature expression and response to AI in treated patients (n=227) for the three response categories using ordinal logistic regression models with OR towards poor response and for the two Ki67_2wks_  categories: high vs low, using univariate logistic regression models with OR towards high· In bold FDR values are the significant signatures. . **Abbreviations**: *ESR1*, Estrogen Receptor 1; *ERBB2*, gene HER2; *FOXA1*, Forkhead BoxA1; HRD, Homologous Recombination Deficiency; *PGR*: Progesterone Receptor*; PD-L1*, Programmed death-ligand 1; Treg, Regulatory T cells; *PTEN*, Phosphatase and tensin homolog*; IDO1*, Indoleamine 2,3-dioxygenase; AR, Androgen Receptor, IFN Gamma, Interferon Gamma; TIS, Tumour Inflammation Signature; *PD-L2*, Programmed death-ligand 2; TIGIT, T cell immunoreceptor with Ig and ITIM domain; APM, Antigen Processing Machinery; BRCAness, Breast Cancer Gene deficiency; *CDK6*, Cyclin-dependant kinase 6; *TGFB*, Transforming growth Factor Beta; *Rb1*, Retinoblastoma; *SOX2*, SRY(Sex determining region Y)-box2; MHC2, major histocompatibility complex 2; *PD1*, Programmed cell death protein 1*; CDK4*, Cyclin-dependant kinase 4; CD8 T cells, Cytotoxic T lymphocytes; HER2-E, HER2-Enriched Subtype; LumB, Luminal B subtype; LumA, Luminal A subtype; OR, Odds Ratio; FDR, False discovery rate, CI, Confidence Interval, Ki67_2w,_ Ki67 at 2 weeks timepoint.

**Supplementary table S4.** Association analysis of signature expression and response to AI in treated patients (n=227) for the two Ki67 response categories: >20% vs <20%, using univariate logistic regression models with OR towards high. **Abbreviations**: *ESR1*, Estrogen Receptor 1; *ERBB2*, gene HER2; *FOXA1*, Forkhead BoxA1; HRD, Homologous Recombination Deficiency; *PGR*: Progesterone Receptor*; PD-L1*, Programmed death-ligand 1; Treg, Regulatory T cells; *PTEN*, Phosphatase and tensin homolog*; IDO1*, Indoleamine 2,3-dioxygenase; AR, Androgen Receptor, IFN Gamma, Interferon Gamma; TIS, Tumour Inflammation Signature; *PD-L2*, Programmed death-ligand 2; TIGIT, T cell immunoreceptor with Ig and ITIM domain; APM, Antigen Processing Machinery; BRCAness, Breast Cancer Gene deficiency; *CDK6*, Cyclin-dependant kinase 6; *TGFB*, Transforming growth Factor Beta; *Rb1*, Retinoblastoma; *SOX2*, SRY(Sex determining region Y)-box2; MHC2, major histocompatibility complex 2; *PD1*, Programmed cell death protein 1*; CDK4*, Cyclin-dependant kinase 4; CD8 T cells, Cytotoxic T lymphocytes; HER2-E, HER2-Enriched Subtype; LumB, Luminal B subtype; LumA, Luminal A subtype; OR, Odds Ratio; FDR, False discovery rate, CI, Confidence Interval, Ki67_2w,_ Ki67 at 2 weeks timepoint.

**Supplementary figure S2**· Differential signature expression by subtypes including HER2-E BC (n=95) and Luminal BC tumours (n=128)· The scatterplots show the logORs from the logistic regression models calculated for the expression of the 46 signatures for a. Ki67 response categories and b. Ki67 2 weeks categories. **Abbreviations**: HER2-E, HER2-Enriched Subtype; LumB, Luminal B subtype; LumA, Luminal A; OR, Odds Ratio.

**Supplementary figure S3·** *ESR1* gene expression levels by PAM50 intrinsic subtype and coloured by different levels (tertiles) of *ERBB2* gene expression. **Abbreviations:** HER2-E, HER2-Enriched Subtype; LumB, Luminal B subtype; LumA, Luminal A subtype; T, Tertiles.

**Supplementary table S5.** Association analysis of the new molecular subgroups based on single gene expression and response to AI by different endpoints. **Abbreviations:** GR, Good Response, IR: Intermediate Response, PR: Poor response.

**Supplementary figure S4.** Differential expression of **A·** Ki67 percentage changes **B·** *ERBB2* and **C·** *ESR1* by the 5 new subgroups in the entire cohort. **Abbreviations**: C, Cluster; HER2-E, HER2-Enriched Subtype; LumB, Luminal B subtype; LumA, Luminal A subtype.

**Supplementary table S6.** Multivariable cox regression analysis for TTR for selected prognosis factors and other variables driving differential adjuvant chemotherapy and HER2 treatment choice. **Abbreviations:** HR, Hazard Ratio; FDR, False discovery rate, CI: Confidence Interval; N, nodal; EMC: Extra celular matrix, HER2-E, HER2-enriched

**Supplementary figure S5.** Barplots comparing the multivariate chi-square likelihood test for each multivariable model. **Abbreviations**: p: p-value; DF: degrees of freedom.

**Supplementary figure S6.** Kaplan Meier curves stratified by adjuvant treatment (trastuzumab + chemotherapy yes vs no) for TTR according to a. PAM50 Intrinsic Subtypes and b. to the new molecular subgroups. **Abbreviations:** HER2-E: HER2-enriched, CT:Chemotherapy, TTR: Time To Recurrence.

**Supplementary table S7·** Series of multivariable analysis for TTR of signature expression adjusted by the basic clinicopathological factors (age, nodal status, post tumour size, post tumour grade). **Abbreviations**: *ESR1*, Estrogen Receptor 1; *ERBB2*, gene HER2; *FOXA1*, Forkhead BoxA1; HRD, Homologous Recombination Deficiency; *PGR*: Progesterone Receptor, BC: Breast Cancer Signature; *PD-L1*, Programmed death-ligand 1; Treg, Regulatory T cells; *PTEN*, Phosphatase and tensin homolog; *IDO1*, Indoleamine 2,3-dioxygenase; *AR*, Androgen Receptor, IFN Gamma, Interferon Gamma; TIS, Tumour Inflammation Signature; *PD-L2*, Programmed death-ligand 2; TIGIT, T cell immunoreceptor with Ig and ITIM domain; APM, Antigen Processing Machinery; BRCAness: Breast Cancer Gene deficiency; *CDK6,* Cyclin-dependant kinase 6; *TGFB*: Transforming growth Factor Beta; *Rb1*, Retinoblastoma; *SOX2*, SRY(Sex determining region Y)-box2; MHC2, major histocompatibility complex 2; *PD1*, Programmed cell death protein 1; *CDK4*, Cyclin-dependant kinase 4; CD8 T cells, Cytotoxic T lymphocytes; HR, Hazard Ratio; FDR, False discovery rate, CI: Confidence Interval.

**Supplementary figure S7.** Spearman correlation tests of the significant signatures by nominal p-value in the series of multivariable analysis using each of the signatures in the baseline clinicopathological model.
